# Nutritional deficiencies in low-sociodemographic-index countries: a population-based study

**DOI:** 10.3389/fnut.2023.985221

**Published:** 2023-04-17

**Authors:** Huansong Li, Huiming Ren, Xu Guo, Zhu Chen

**Affiliations:** ^1^Department of Rehabilitation Medicine, Ningbo No.2 Hospital, Ningbo, China; ^2^Department of Laboratory, Ningbo No.2 Hospital, Ningbo, China

**Keywords:** nutritional deficiencies, low-sociodemographic-index, incidence, disability-adjusted life-years (DALYs), Global Burden of Diseases

## Abstract

**Background:**

We aimed to estimate the burden of nutritional deficiency according to sex and age in countries with a low sociodemographic index (SDI).

**Methods:**

Following the methods of the Global Burden of Diseases, Injuries, and Risk Factors Study 2019, estimated annual percentage changes (EAPCs) were calculated to determine trends in the age-standardized rates of incidence and disability-adjusted life-years (DALYs) of nutritional deficiency and its main subcategories from 1990 to 2019 in low-SDI countries.

**Findings:**

From 1990 to 2019, the age-standardized incidence and DALY rates of nutritional deficiency showed decreasing trends, with EAPCs of −0.90 [95% confidence interval (CI), 1.06 to −0.75] and −3.20 (95% CI, −3.29 to −3.10), respectively, in low-SDI countries. In 2019, of the subcategories analyzed, vitamin A deficiency had the highest age-standardized incidence rate and protein–energy malnutrition had the highest age-standardized DALY rate. From 1990 to 2019, the greatest decrease in the age-standardized incidence rate was observed for vitamin A deficiency and the greatest decrease in the age-standardized DALY rate was observed for protein–energy malnutrition. At the national level, from 1990 to 2019, the greatest increase in the age-standardized incidence rate of overall nutritional deficiency was observed in males in Afghanistan (EAPC: 0.28; 95% CI, 0.07 to 0.49). Of the age groups analyzed, the highest incidence and DALY rates of overall nutritional deficiency and dietary iron deficiency were observed in children aged 1–4 years.

**Interpretation:**

The age-standardized incidence and DALY rates of nutritional deficiency decreased significantly from 1990 to 2019, especially for vitamin A deficiency and protein–energy malnutrition. Overall nutritional deficiency and dietary iron deficiency occurred primarily in children aged 1–4 years.

## Introduction

Insufficient nutrient intake or nutritional deficiency, termed “hidden hunger” by the World Health Organization (WHO) (1, 2), has a major effect on physical and mental health. Children and adolescents are two groups that are most affected by hidden hunger because of their high demand for nutrient intake (3, 4). Moreover, many school students have problems with memory, concentration, immunity, mood, and growth and development due to nutritional deficiency (5, 6). Nutritional deficiencies may also lead to associated diseases and increase the risk of chronic non-communicable diseases (7).

Despite progress in achieving the United Nations Decade of Action on Nutrition 2016–2025 goals and the Sustainable Development Goal to “end all forms of malnutrition,” nutritional deficiencies remain prevalent in countries that have a low sociodemographic index (SDI). In low-SDI countries, inadequate intake of nutrients caused by insufficient food quantity and unhealthy dietary structure is the main reason for nutritional deficiencies (8). Due to interactions between various nutrients, the prevalent nutritional deficiency is usually not of a single nutrient but rather a deficiency of multiple nutrients (9). Among the subcategories of nutritional deficiency, vitamin A deficiency remains widespread in South Asia and Sub-Saharan Africa (10), while the iodine deficiency rate is greater than 50% in preschool children of 13 African countries (11).

This study aimed to provide a comprehensive estimate of the incidence of nutritional deficiency and its main subcategories at the global level, and at the national level in low-SDI countries, using data collected from 204 countries and territories in the Global Burden of Diseases, Injuries, and Risk Factors Study (GBD) 2019.

## Materials and methods

### Overview

The GBD 2019 data set comprises epidemiological data from all up-to-date sources, and the study used improved standardized methods to estimate the incidence, prevalence, mortality, years of life lost (YLLs), years lived with disability (YLDs), and disability-adjusted life-years (DALYs) for 369 diseases and injuries in both sexs and in 204 countries and territories, including 33 low-SDI countries. Hwa Mei Hospital reviewed and approved the present study.

### Data sources

The original nutritional deficiency data estimated by the GBD 2019 were obtained mainly from censuses, household surveys, civil registration and vital statistics, disease registries, health service use, air pollution monitors, satellite images, disease notifications, and other sources (12).

Nutritional deficiencies were identified based on the criteria listed in the 10th revision of the International Classification of Diseases and Injuries and included protein–energy malnutrition, iodine deficiency, vitamin A deficiency, dietary iron deficiency, and other nutritional deficiencies (Supplementary Table 1).

### Estimation of incidence and DALYs

Two measures were extracted for this study: incidence and DALYs. Age-standardized rates (ASRs) and estimated annual percentage changes (EAPCs) were calculated for these two measures to quantify the trends in these measures. The incidence and DALY data for different age groups were also extracted to quantify the burden of nutritional deficiency. The reported age-standardized incidence rates correspond to the number of cases per 100,000 persons, and the reported age-standardized DALY rates correspond to the number of YLDs and the number of YLLs per 100,000 persons after age standardization. DALYs were calculated by summing the YLLs and YLDs, thereby incorporating both the fatal and non-fatal burdens.

### Statistical analysis

Estimated annual percentage changes is a quantitative index that is widely used to estimate the annual average change in the ASR of any disease burden indicator in a specific time interval and thus reflects the trend in the ASR (13). Each observation value contributes to an EAPC, in the form of the natural logarithm of the time variable and its corresponding observation value. Moreover, an EAPC estimates and quantifies the long-term trend in disease burden indicators such as the incidence and mortality of diseases. Thus, an EAPC can not only evaluate the rate of change in these indicators but also predict their future trends. Therefore, by analyzing an ASR and an EAPC, the effectiveness of current disease prevention strategies can be established, and more targeted strategies can be formulated when necessary. When calculating an EAPC based on an ASR, the calendar year is an independent variable, and the natural logarithm of the ASR is analyzed by linear regression.

The EAPC in this study was calculated as previously described (13) using the following formulae: y = a + bx + ∈ and EAPC = 100 × [exp (β) − 1], where y = ln(ASR), x is the calendar year, and β is the estimated value of the slope b. The EAPC formula was then used to calculate the 95% confidence interval (CI), and the standard error was obtained from the fitted regression line. If the estimated EAPC and the lower boundary of its 95% CI were both > 0, the ASR was considered to have an increasing trend. In contrast, if the estimation of the EAPC and the upper boundary of its 95% CI were both < 0, the ASR was considered to have a decreasing trend. Otherwise, the ASR was considered to be stable over time. The analyses were performed using R version 3.3.

## Results

### Analysis of the incidence of nutritional deficiency in low-SDI countries

**FIGURE 1:**
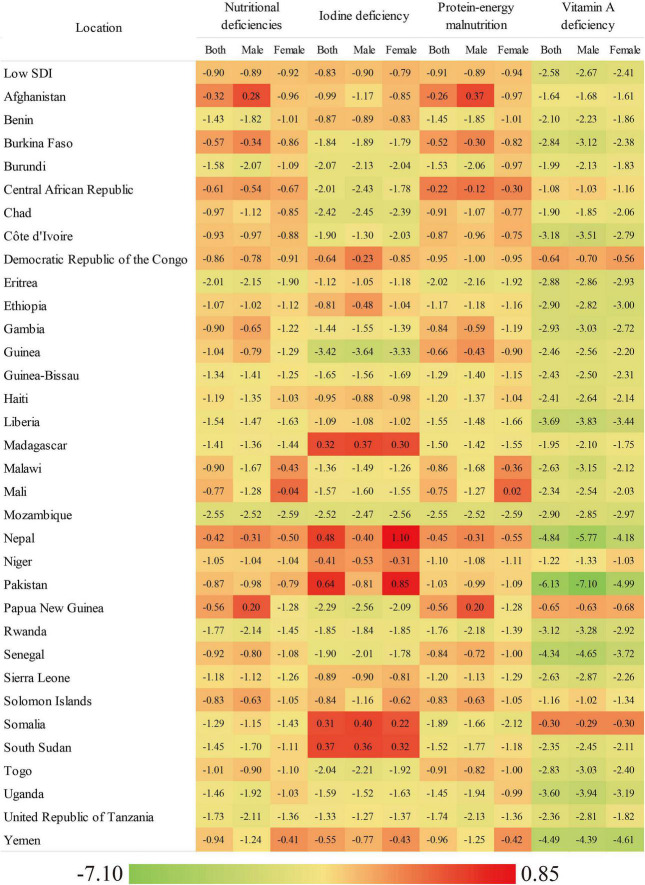
The EAPC of age-standardized incidence rate of overall nutritional deficiency and its main subcategories from 1990 to 2019, by gender and country.

From 1990 to 2019, the global age-standardized incidence rate of nutritional deficiency remained stable in males but showed a consistent decreasing trend in females, with an EAPC of −0.23 (Table 1). Overall, in 2019, the number of incident cases of overall nutritional deficiency was 2250.16 per 100,000 in males and 2056.49 per 100,000 and in females in low-SDI countries. From 1990 to 2019, the age-standardized incidence rate showed a consistent decreasing trend in both males and females, with an EAPC of −0.89 and −0.92, respectively, in low-SDI countries (Figure 1 and Table 1).

**TABLE 1:** Age-standardized incidence rates for nutritional deficiency (including subcategories) and their estimated annual percentage changes (EAPC) in low-and middle-income countries (LMIC) by sex group, 1990–2019.

	Incidence			DALY		
	**1990**	**2019**	**EAPC**	**1990**	**2019**	**EAPC**
**Global**						
Nutritional deficiencies	2025.70 (1691.30, 2430.98)	2207.71 (1863.04, 2604.67)	−0.05 (−0.20,0.09)	1496.94 (1186.80,1903.48)	680.12 (507.21, 894.89)	−2.91 (−3.05, −2.76)
Protein-energy malnutrition	1896.61 (1563.69, 2293.12)	2099.39 (1752.77, 2487.42)	−0.03 (−0.19, 0.13)	855.25 (654.33, 1138.19)	218.29 (179.53, 262.77)	−5.03 (−5.28, −4.77)
Iodine deficiency	129.09 (105.20, 156.72)	108.32 (86.83,1 33.34)	−0.44 (−0.58, −0.31)	46.85 (28.65,76.01)	30.70 (17.32,53.13)	−1.35 (−1.45, −1.25)
Vitamin A deficiency	17323.23 (16526.51, 18138.92)	6955.65 (6645.87, 7294.23)	−3.11 (−3.25, −2.98)	31.95 (22.11, 45.30)	16.91 (11.53, 23.47)	−2.18 (−2.38, −1.98)
Dietary iron deficiency	0.00 (0.00, 0.00)	0.00 (0.00, 0.00)	-	458.54 (308.85, 657.39)	383.38 (257.05, 553.46)	−0.64 (−0.67, −0.61)
Other nutritional deficiencies	0.00 (0.00, 0.00)	0.00 (0.00, 0.00)	-	104.36 (66.23, 144.64)	30.84 (24.65, 3 8.32)	−4.35 (−4.69, −4.01)
**High-middle SDI**						
Nutritional deficiencies	1427.42 (1160.31, 1756.34)	1879.06 (1540.14, 2291.77)	0.76 (0.62, 0.91)	511.96 (386.08, 679.39)	275.47 (189.75, 384.60)	−2.38 (−2.50, −2.26)
Protein-energy malnutrition	1365.91 (1091.94, 1696.29)	1827.94 (1489.86, 2234.50)	0.82 (0.67, 0.97)	156.44 (134.24, 183.94)	64.02 (46.09, 85.06)	−3.43 (−3.72, −3.14)
Iodine deficiency	61.50 (49.92, 75.77)	51.12 (40.65, 63.41)	−0.68 (−0.83, −0.54)	18.47 (10.41, 32.05)	13.48 (6.71, 25.34)	−1.30 (−1.46, −1.14)
Vitamin A deficiency	7634.98 (7130.23, 8221.88)	2624.49 (2484.54, 2776.44)	−3.71 (−3.81, −3.62)	9.28 (6.17, 13.25)	3.74 (2.49, 5.43)	−3.29 (−3.38, −3.21)
Dietary iron deficiency	0.00 (0.00, 0.00)	0.00 (0.00, 0.00)	–	303.72 (200.79, 439.78)	175.57 (114.85, 261.53)	−2.13 (−2.23, −2.04)
Other nutritional deficiencies	0.00 (0.00, 0.00)	0.00 (0.00, 0.00)	–	24.06 (18.47, 30.85)	18.65 (13.14, 25.74)	−0.79 (−1.12, −0.45)
**High SDI**						
Nutritional deficiencies	912.00 (749.28, 1100.80)	1062.62 (870.67, 1295.90)	0.19 (0.06, 0.31)	187.65 (128.93, 263.33)	130.80 (90.96, 180.72)	−1.28 (−1.47, −1.09)
Protein-energy malnutrition	889.73 (729.16, 1080.56)	1042.08 (847.01, 1278.09)	0.20 (0.07, 0.33)	38.75 (28.96,51.17)	40.03 (28.18, 55.03)	−0.19 (−0.29, −0.08)
Iodine deficiency	22.27 (17.68, 28.06)	20.54 (16.37, 25.80)	−0.29 (−0.30, −0.27)	4.62 (2.14, 8.84)	4.14 (1.90, 7.94)	−0.38 (−0.39, −0.38)
Vitamin A deficiency	1338.23 (1268.38, 1413.80)	586.69 (550.35, 625.85)	−2.69 (−2.78, −2.61)	0.91 (0.58, 1.35)	0.29 (0.18, 0.44)	−3.49 (−3.94, −3.04)
Dietary iron deficiency	0.00 (0.00, 0.00)	0.00 (0.00, 0.00)	–	124.36 (80.58, 183.61)	72.61 (46.01, 112.29)	−1.84 (−2.11, −1.57)
Other nutritional deficiencies	0.00 (0.00, 0.00)	0.00 (0.00, 0.00)	–	19.02 (13.11, 26.77)	13.73 (9.24, 19.53)	−0.82 (−1.38, −0.25)
**Low-middle SDI**						
Nutritional deficiencies	2840.71 (2414.99, 3355.44)	2707.55 (2289.16, 3191.73)	−0.68 (−0.84, −0.52)	2779.35 (2058.88, 3664.31)	946.24 (697.13, 1264.18)	−3.98 (−4.27, −3.70)
Protein-energy malnutrition	2600.21 (2169.98, 3101.03)	2560.79 (2142.30, 3030.93)	−0.61 (−0.79, −0.43)	1552.95 (1055.42, 2209.13)	222.78 (183.77, 268.24)	−7.28 (−7.84, −6.72)
Iodine deficiency	240.50 (197.32, 291.39)	146.76 (114.94, 184.41)	−1.55 (−1.86, −1.24)	107.22 (67.51, 171.95)	50.55 (29.32, 86.65)	−2.37 (−2.52, −2.22)
Vitamin A deficiency	26646.41 (25022.20, 28112.34)	8475.93 (7893.16, 9132.39)	−3.95 (−4.13, −3.76)	50.95 (35.19, 72.27)	19.32 (12.93, 27.45)	−3.38 (−3.60, −3.16)
Dietary iron deficiency	0.00 (0.00, 0.00)	0.00 (0.00, 0.00)	–	767.72 (519.77, 1084.10)	605.09 (408.83, 867.44)	−0.80 (−0.85, −0.75)
Other nutritional deficiencies	0.00 (0.00, 0.00)	0.00 (0.00, 0.00)	–	300.52 (146.16, 475.33)	48.50 (39.57, 58.95)	−6.43 (−6.88, −5.97)
**Low SDI**						
Nutritional deficiencies	2513.76 (2162.93, 2910.72)	2153.30 (1866.44, 2483.55)	−0.90 (−1.06, −0.75)	3412.28 (2774.81, 4306.92)	1412.48 (1090.05, 1802.47)	−3.20 (−3.29, −3.10)
Protein-energy malnutrition	2245.57 (1901.58, 2655.55)	1948.29 (1670.53, 2280.57)	−0.91 (−1.08, −0.74)	2212.47 (1717.40, 2942.81)	524.59 (422.09, 652.20)	–5.13 (−5.28, −4.98)
Iodine deficiency	268.19 (225.04, 320.24)	205.01 (167.11, 248.21)	−0.83 (−0.98, −0.69)	111.19 (67.54, 184.90)	78.06 (45.66, 133.80)	−1.16 (−1.36, −0.97)
Vitamin A deficiency	37932.60 (36877.72, 38931.64)	18004.56 (17394.92, 18637.14)	–2.58 (−2.80, −2.36)	73.54 (50.66, 102.90)	38.02 (26.12, 52.78)	−2.30 (−2.50, −2.11)
Dietary iron deficiency	0.00 (0.00, 0.00)	0.00 (0.00, 0.00)	–	800.65 (540.70, 1139.77)	714.22 (481.51, 1030.49)	−0.43 (−0.46, −0.39)
Other nutritional deficiencies	0.00 (0.00, 0.00)	0.00 (0.00, 0.00)	–	214.43 (145.65, 302.35)	57.57 (46.49, 69.62)	−4.86 (−5.11, −4.60)
**Middle SDI**						
Nutritional deficiencies	1924.12 (1579.34, 2347.44)	2223.00 (1846.85, 2643.85)	0.23 (0.09, 0.37)	925.96 (741.71, 1164.77)	445.72 (320.11, 601.15)	−2.56 (−2.69, −2.43)
Protein-energy malnutrition	1839.82 (1491.32, 2265.01)	2153.13 (1778.80, 2566.20)	0.26 (0.12, 0.41)	388.87 (337.54, 455.73)	118.65 (96.81, 142.93)	−4.15 (−4.33, −3.97)
Iodine deficiency	84.30 (67.43, 104.35)	69.86 (55.41, 87.18)	−0.61 (−0.70, −0.52)	35.61 (21.90, 55.95)	19.67 (10.35, 36.02)	−2.09 (−2.18, −2.00)
Vitamin A deficiency	12892.40 (12128.34, 13703.92)	3679.01 (3464.06, 3921.19)	−4.16 (−4.25, −4.07)	21.53 (14.48, 30.75)	8.61 (5.75, 12.42)	−3.14 (−3.23, −3.05)
Dietary iron deficiency	0.00 (0.00, 0.00)	0.00 (0.00, 0.00)	—	421.88 (282.19, 607.47)	273.75 (182.59, 390.97)	−1.54 (−1.62, −1.47)
Other nutritional deficiencies	0.00 (0.00, 0.00)	0.00 (0.00, 0.00)	–	58.07 (47.01, 69.85)	25.04 (19.24, 32.14)	−2.94 (−3.19, −2.69)

DALYs, disability-adjusted life-years.

In low-SDI countries, of the nutritional deficiency subcategories analyzed, vitamin A deficiency had the highest age-standardized incidence rate in males (21,850.89) and females (14,190.34) in 2019, followed by protein–energy malnutrition in males (2,098.69) and females (1,797.70) and iodine deficiency in males (151.47) and females (258.79) (Table 1). However, from 1990 to 2019, the age-standardized incidence rate of vitamin A deficiency showed the greatest decrease in males (EAPC: −2.67) and females (EAPC: −2.41), and the age-standardized incidence rate of iodine deficiency showed the smallest decrease (Table 1).

Figure 1 and Supplementary Table 2 present the EAPCs in the age-standardized incidence rates of overall nutritional deficiency and its main subcategories stratified by sex for low-SDI countries from 1990 to 2019. In 2019, of all of the nutritional deficiency subcategories analyzed in low-SDI countries, the age-standardized incidence rate of vitamin A deficiency was highest (females: 14,190.34, males: 21,850.89), followed by those of protein–energy malnutrition (females: 1,797.70, males: 2,098.69) and iodine deficiency (females: 258.79, males: 151.47; Supplementary Table 2). From 1990 to 2019, the greatest decrease in the age-standardized incidence rate was observed for vitamin A deficiency. This was observed in both sexs and in almost all of the low-SDI countries evaluated (Supplementary Table 2).

At the national level in 2019, the age-standardized incidence rate of overall nutritional deficiency in females ranged from 667.97 in Mozambique to 2,575.77 in Pakistan. In males, this rate ranged from 751.83 in Uganda to 3,195.57 in Yemen (Supplementary Table 2). From 1990 to 2019, the age-standardized incidence rate of nutritional deficiency decreased in females in all of the countries analyzed and increased in males in only two countries, with the greatest increase seen in males in Afghanistan (EAPC: 0.28). Moreover, the greatest decreases in the age-standardized incidence rate of nutritional deficiency in females and males were seen in Mozambique (EAPC: −2.59 and −2.52, respectively; Supplementary Table 2).

In 2019, the highest age-standardized incidence rate was observed for vitamin A deficiency in both sexes in all of the low-SDI countries analyzed. This was especially true for Somalia, which had the highest age-standardized incidence rates of vitamin A deficiency (females: 54,280.58, males: 72,502.08; Supplementary Table 2). From 1990 to 2019, the age-standardized incidence rates for all of the nutritional deficiency subcategories decreased in the majority of low-SDI countries. In addition, the greatest decrease in the age-standardized incidence rate was observed for vitamin A deficiency in both sexs and in almost all of the countries evaluated (Table 1). The age-standardized incidence rate of protein–energy malnutrition increased in females in one country and in males in two countries, with males in Afghanistan showing the greatest increase (EAPC: 0.37). The age-standardized incidence rate of iodine deficiency increased in females in five countries and in males in three countries, with females in Nepal (EAPC: 1.10) and males in Somalia (EAPC: 0.40) showing the greatest increases (Supplementary Table 2).

**FIGURE 2:**
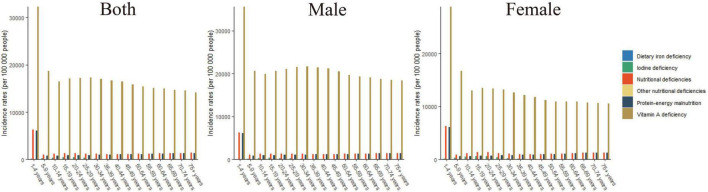
The incidence rates of overall nutritional deficiency and its main subcategories by age groups in 2019.

Of the different age groups analyzed, the highest incidence rates of overall nutritional deficiency, vitamin A deficiency, and dietary iron deficiency were observed in children aged 1–4 years (Figure 2). Of the nutritional deficiency subcategories analyzed, the highest incidence rate was observed for vitamin A deficiency in all of the age groups.

### DALY

**FIGURE 3:**
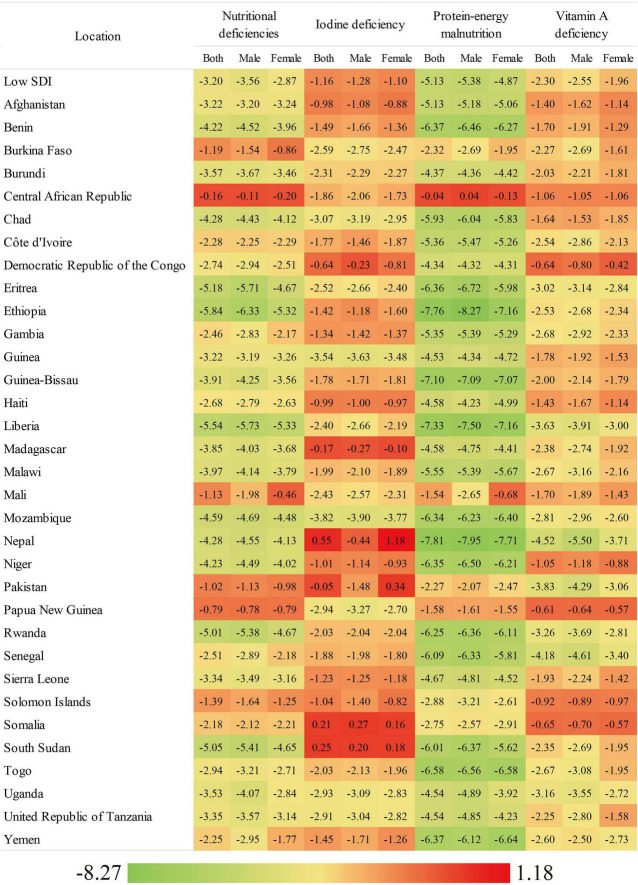
The EAPC of age-standardized DALYs rate of overall nutritional deficiency and its main subcategories from 1990 to 2019, by gender and country.

Globally, the number of DALYs associated with overall nutritional deficiency was 49,775,123.92 per 100,000 in 2019. From 1990 to 2019, the global age-standardized number of DALYs showed a consistently decreasing trend, with an EAPC of −2.91 (Figure 3 and Table 1).

Overall, in 2019, the number of DALYs associated with new cases of overall nutritional deficiency was 1,412.48 per 100,000 in low-SDI countries. From 1990 to 2019, the age-standardized DALY rate showed a consistent decreasing trend (EAPC: −3.20) in low-SDI countries (Figure 3 and Table 1). However, from 1990 to 2019, the age-standardized DALY rate associated with protein–energy malnutrition showed the greatest decrease (EAPC: −5.13), while that associated with dietary-iron deficiency showed the smallest decrease (EAPC: −0.43; Table 1).

Of all of the nutritional deficiency subcategories analyzed in low-SDI countries in 2019, the age-standardized DALY rate was highest for protein–energy deficiency (females: 549.51, males: 500.41), followed by iodine deficiency (females: 94.65, males: 61.23) and vitamin A deficiency (females: 33.87, males: 42.03; Supplementary Table 3). From 1990 to 2019, the greatest decrease in the age-standardized DALY rate was observed for protein–energy deficiency in both sexs and in almost all of the low-SDI countries evaluated (Supplementary Table 3).

However, in 2019, the age-standardized DALY rate was highest for iodine deficiency in both sexs, especially in Somalia, which had the highest age-standardized DALY rate for iodine deficiency (females: 300.16, males: 247.34; Supplementary Table 3). The age-standardized DALY rates for all of the subcategories decreased from 1990 to 2019, with the greatest decreases observed for protein–energy deficiency in males in Ethiopia (EAPC: −8.27) and females in Nepal (EAPC: −7.71), followed by vitamin A deficiency in both males (EAPC: −5.50) and females (EAPC: −3.71) in Nepal (Supplementary Table 3).

At the national level in 2019, the age-standardized DALY rate for overall nutritional deficiency in females ranged from 722.16 per 100,000 in Afghanistan to 5,442.08 per 100,000 in Mali. In males, this rate ranged from 372.81 per 100,000 in Afghanistan to 3,345.93 per 100,000 in Somalia. The greatest sex gap was observed in Mali (female/male ratio approximately 1.7; Supplementary Table 3). From 1990 to 2019, the age-standardized DALY rate for overall nutritional deficiency decreased in almost all of the countries evaluated, with the greatest decreases observed in females in Liberia (EAPC: −5.33) and in males in Ethiopia (EAPC: −6.33; Supplementary Table 3).

In 2019, the age-standardized DALY rate was highest for protein–energy malnutrition in all of the low-SDI countries analyzed, and highest in females in Mali (4,163.90) and in males in Somalia (2,355.54; Supplementary Table 3). The age-standardized DALY rates for all of the nutritional deficiency subcategories decreased in the majority of low-SDI countries from 1990 to 2019. However, for iodine deficiency, the age-standardized DALY rate increased in females in four countries and in males in two countries, with Nepal showing the greatest increase in females (EAPC: 1.18) and Somalia showing the greatest increase in males (EAPC: 0.27). For iron deficiency, the age-standardized DALY rates decreased in most of the countries, especially in females (EAPC: −3.71) and males (EAPC: −5.50) in Nepal (Supplementary Table 3).

**FIGURE 4:**
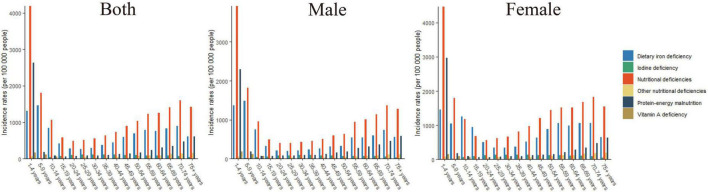
The DALYs rates of overall nutritional deficiency and its main subcategories by age groups in 2019.

Of the different age groups analyzed, the highest DALY rates for overall nutritional deficiency, protein–energy malnutrition, and dietary iron deficiency were observed in children below the age of 5 (Figure 4). Of the nutritional deficiency subcategories analyzed, the highest DALY rate was observed for protein–energy malnutrition in all of the age groups.

## Discussion

We systematically evaluated the burden of nutritional deficiency in low-SDI countries and found that its age-standardized incidence and DALY rates decreased from 1990 to 2019. During this period, the age-standardized incidence rate of vitamin A deficiency and the age-standardized DALY rate for protein–energy malnutrition showed the greatest decreases of all of the nutritional deficiency subcategories analyzed.

The decreasing trends in the age-standardized incidence and DALY rates of nutritional deficiency in low-SDI countries from 1990 to 2019 are probably attributable to improvements in health service systems, breastfeeding rates, complementary feeding, immunization coverage, water and sanitation measures, and food fortification measures (14). However, rapid urbanization has affected dietary patterns, with a shift toward energy-dense but low-nutrient fast foods, which are contributing to the double burden of undernutrition and overweight (15). Dietary patterns in low-SDI countries have undergone great changes, as eating out and snack consumption are becoming increasingly common (16, 17), resulting in a dietary structure that is high in fat, low in carbohydrates, and deficient in micronutrients (18). Thus, addressing nutritional deficiencies in low-SDI countries requires combining strategies that address undernutrition and dietary diversity.

A key finding is that from 1990 to 2019, the age-standardized incidence rates of overall nutritional deficiency and the protein–energy malnutrition subcategory showed the greatest increases in males in Afghanistan. This could be attributable to the war in Afghanistan that has led to a lack of safe water and sanitation, poor quality housing, poor nutrition, and limited access to quality health services (19, 20).

Of the nutritional deficiency subcategories, the age-standardized incidence rate of vitamin A deficiency was highest in 2019, but it showed the greatest decrease from 1990 to 2019 in both sexs and in almost all of the low-SDI countries. The age-standardized DALY rate for vitamin A deficiency also decreased in this period. The WHO recognizes vitamin A deficiency as one of the four major nutritional deficiencies globally (21). There is a basic understanding of the adverse effects of vitamin A deficiency (clinical and subclinical), and the main reasons for vitamin A deficiency are clear (22). Increasing the variety and quantity of animal- and plant-based foods rich in vitamin A and carotene in the daily diet and consuming large doses of vitamin A supplements are strategies that may reduce the prevalence of vitamin A deficiency in economically underdeveloped areas and major affected areas (23). In addition, it is recommended that serum vitamin A concentrations of children younger than 6 years (especially those younger than 3 years) are tested yearly (24).

From 1990 to 2019, the age-standardized incidence and DALY rates for iodine deficiency showed the greatest increases in females in Nepal and in males in Somalia. Thus, these countries should take various measures to improve the iodine nutrition status of their populations, promote dietary diversification, and fortify bread, milk, water, and salt with iodine. Because salt iodization is effective, safe, simple to execute, and inexpensive, salt iodization as an iodine deficiency prevention and control measure is currently adopted in many countries.

Of the age groups analyzed, the highest incidence and DALY rates of overall nutritional deficiency and dietary iron deficiency were observed in children aged 1–4 years. The highest incidence rate of vitamin A deficiency was also observed in children aged 1–4 years. Lack of access to a sufficient quantity of food, inadequate food intake, poor breastfeeding, limited access to health services, and diseases lead to malnutrition in children. In the US, the prevalence of anemia and vitamin D deficiency in the population aged 9 years and above was found to be over 6% from National Health and Nutrition Examination Survey (NHANES) 2007–2010) (25). Several vitamin A deficiency prevention and treatment strategies, including mass and point-of-use food fortification, dietary diversification, and periodic (4- to 6-monthly) high-dose supplementation with vitamin A capsules or tablets (26), have contributed to the decrease in vitamin A deficiency in children, but “this has taken several years to occur.”

The age-standardized incidence rate of overall nutritional deficiency was higher in males than in females in 2019. The increased susceptibility of males to nutritional deficiency may be due to their having a higher energy demand and higher protein metabolism index than females (27), apathy toward maintaining a healthy diet (28), frequent exercise-accelerated nutrient consumption, smoking, alcohol consumption, drug use, and acquired immunodeficiency syndrome-accelerated nutrient loss (29).

The factors influencing nutritional deficiency include sex, age, per capita monthly income level, attitude toward nutrition knowledge, and attitude toward nutritional supplements and demand (30). Our findings suggest the need to (i) pay special attention to the rational use of nutrient supplements for people in special occupations or special environments and (ii) strengthen the guidance on the use of nutrient supplements, especially by clarifying that nutrient supplements are dietary supplements that cannot replace meals, that the role of nutrient supplements in preventing cardiovascular and cerebrovascular diseases is ambiguous, and that some nutrient supplements may be harmful to health if taken for a prolonged period. Therefore, it is recommended that people consult professionals before taking nutrient supplements, to determine the rationality of their use.

To the best of our knowledge, this is the first comprehensive overview and report of the burden of nutritional deficiency in low-SDI countries and its associated incidence rates, DALY rates, and EAPCs. This study also identified high-risk populations through sex and age stratification. However, this study has one main limitation. Although the GBD 2019 adjusted for low-quality sampling, survey methods, and other methodological deficiencies, the accuracy of the nutritional deficiency analysis largely depends on the quality and quantity of the data input into the models.

## Conclusion

The age-standardized incidence and DALY rates of nutritional deficiency decreased significantly from 1990 to 2019, especially for vitamin A deficiency. The age-standardized DALY rate for overall nutritional deficiency decreased in both sexs, with the most pronounced decreases observed for protein–energy malnutrition. Among all of the age groups, overall nutritional deficiency and dietary iron deficiency were concentrated in children aged 1–4 years in the study period.

## Data availability statement

The raw data supporting the conclusions of this article will be made available by the authors, without undue reservation.

## Author contributions

HR conceived the idea for this initiative. HL and XG contributed to reading the literature, preparation of figures and the table, and writing the manuscript. ZC assisted with the writing and revising the manuscript. All authors read and approved the final manuscript.
