# Supplementary material for: Nutritional deficiencies in low-sociodemographic-index countries: a population-based study
Source: Front Nutr. 2023 Apr 17;10:985221. doi: 10.3389/fnut.2023.985221 (PMC10149740; doi:10.3389/fnut.2023.985221)
Supplement: Supplementary file 1 [file Data_Sheet_1.docx]

#### Table 1 List of International Classification of Diseases (ICD) 10 codes mapped to the Global Burden of Disease cause list for Nutritional deficiency

| Cause | ICD10 |
| --- | --- |
| Nutritional deficiency | E43, E44 -E44.1, E45, E46, E64-E64.9, E00-E00.9, E01-E01.8, E02, E61.8,E50-E50.9, E53-E53.9, E64.1, L86, H19.8, H16.2, H13.8, H58.1, E61.1, D50-50.9,E64, E64.8 |
| protein-energy malnutrition | E43, E44 -E44.1, E45, E46, E64-E64.9 |
| iodine deficiency | E00-E00.9, E01-E01.8, E02, E61.8 |
| vitamin A deficiency | E50-E50.9, E53-E53.9, E64.1, L86, H19.8, H16.2, H13.8, H58.1 |
| dietary iron deficiency | E61.1, D50-50.9 |
| other nutritional deficiency | E64, E64.8 |

**Table 2 Country‐specific age-standardized incidence rate for nutritional deficiency (including Subcategories) and their estimated annual percentage changes (EAPC) in low-sociodemographic-index by sex group, 1990-2019.**

|  | male |  |  | female |  |  |
| --- | --- | --- | --- | --- | --- | --- |
|  | 1990 | 2019 | eapc | 1990 | 2019 | eapc |
| Low SDI |  |  |  |  |  |  |
| Nutritional deficiencies | 2632.81(2260.49,3054.08) | 2250.16(1925.84,2616.74) | -0.89(-1.04,-0.73) | 2393.39(2052.68,2809.53) | 2056.49(1794.09,2356.25) | -0.92(-1.07,-0.76) |
| Protein-energy malnutrition | 2419.91(2052.40,2825.28) | 2098.69(1779.70,2464.17) | -0.89(-1.04,-0.73) | 2069.87(1745.63,2466.92) | 1797.70(1544.57,2084.78) | -0.94(-1.12,-0.76) |
| Iodine deficiency | 212.90(175.64,254.80) | 151.47(122.61,185.78) | -0.90(-1.12,-0.69) | 323.52(271.64,385.91) | 258.79(212.05,314.59) | -0.79(-0.91,-0.68) |
| Vitamin A deficiency | 47234.51(45411.77,49114.17) | 21850.89(20800.45,23005.13) | -2.67(-2.86,-2.48) | 28510.43(27582.08,29549.67) | 14190.34(13655.85,14821.74) | -2.41(-2.67,-2.14) |
| Papua New Guinea |  |  |  |  |  |  |
| Nutritional deficiencies | 1708.17(1441.62,2050.52) | 1815.49(1530.83,2164.69) | 0.20(-0.23,0.62) | 2235.49(1786.95,2851.24) | 1665.85(1288.25,2126.37) | -1.28(-1.49,-1.07) |
| Protein-energy malnutrition | 1702.04(1436.44,2043.17) | 1812.40(1528.56,2162.22) | 0.20(-0.22,0.63) | 2227.28(1778.86,2843.00) | 1661.02(1282.71,2121.04) | -1.28(-1.49,-1.07) |
| Iodine deficiency | 6.14(4.61,8.02) | 3.10(2.24,4.11) | -2.56(-2.95,-2.18) | 8.20(6.21,10.63) | 4.83(3.57,6.37) | -2.09(-2.43,-1.74) |
| Vitamin A deficiency | 18540.09(14089.02,23251.41) | 12943.64(9630.96,17218.26) | -0.63(-1.08,-0.18) | 14855.14(12006.24,18136.72) | 10440.79(8439.50,12971.82) | -0.68(-1.05,-0.31) |
| Solomon Islands |  |  |  |  |  |  |
| Nutritional deficiencies | 1430.30(1196.67,1719.51) | 1298.45(1097.34,1512.77) | -0.63(-0.75,-0.51) | 1581.61(1301.91,1933.71) | 1232.19(1051.71,1441.00) | -1.05(-1.19,-0.90) |
| Protein-energy malnutrition | 1426.11(1193.24,1716.22) | 1295.49(1094.44,1509.45) | -0.63(-0.75,-0.51) | 1576.04(1295.44,1928.69) | 1227.60(1047.86,1436.34) | -1.05(-1.19,-0.90) |
| Iodine deficiency | 4.19(3.08,5.56) | 2.96(2.11,3.93) | -1.16(-1.35,-0.97) | 5.57(4.19,7.26) | 4.59(3.39,6.05) | -0.62(-0.75,-0.49) |
| Vitamin A deficiency | 49032.94(41576.49,56363.48) | 33421.14(26527.57,40515.86) | -1.02(-1.23,-0.82) | 35501.04(29972.74,41420.73) | 22321.01(18216.54,26928.80) | -1.34(-1.55,-1.14) |
| Haiti |  |  |  |  |  |  |
| Nutritional deficiencies | 1477.58(1189.44,1839.94) | 1085.57(904.11,1300.17) | -1.35(-1.51,-1.18) | 1275.65(967.26,1673.56) | 1084.76(888.60,1329.17) | -1.03(-1.17,-0.90) |
| Protein-energy malnutrition | 1424.26(1134.48,1793.09) | 1042.53(860.91,1260.43) | -1.37(-1.53,-1.20) | 1204.90(894.25,1606.60) | 1028.96(833.00,1277.60) | -1.04(-1.18,-0.90) |
| Iodine deficiency | 53.32(40.68,66.85) | 43.04(32.47,55.57) | -0.88(-1.15,-0.60) | 70.75(54.48,87.08) | 55.80(42.14,70.23) | -0.98(-1.26,-0.69) |
| Vitamin A deficiency | 29824.35(23840.57,36411.14) | 14516.80(11144.56,18335.76) | -2.64(-2.70,-2.57) | 23315.42(19643.92,27670.25) | 12558.67(10229.20,15078.79) | -2.14(-2.19,-2.10) |
| Yemen |  |  |  |  |  |  |
| Nutritional deficiencies | 4406.59(3550.90,5460.96) | 3195.57(2623.52,3855.42) | -1.24(-1.37,-1.10) | 2465.62(1894.40,3228.49) | 2025.23(1695.00,2438.58) | -0.41(-0.57,-0.26) |
| Protein-energy malnutrition | 4325.00(3471.30,5378.27) | 3108.97(2546.48,3769.42) | -1.25(-1.39,-1.11) | 2347.96(1783.68,3104.95) | 1888.46(1565.81,2289.70) | -0.42(-0.59,-0.25) |
| Iodine deficiency | 81.59(63.84,100.69) | 86.61(66.85,108.45) | -0.77(-1.25,-0.29) | 117.66(92.36,146.00) | 136.77(106.42,171.79) | -0.43(-0.91,0.06) |
| Vitamin A deficiency | 52553.72(45016.97,60370.66) | 18416.56(13954.13,23369.90) | -4.39(-4.69,-4.09) | 36291.01(31098.33,41353.06) | 11609.73(9212.93,14352.35) | -4.61(-4.95,-4.27) |
| Afghanistan |  |  |  |  |  |  |
| Nutritional deficiencies | 1107.07(908.01,1339.07) | 1377.48(1166.85,1613.83) | 0.28(0.07,0.49) | 1180.44(970.32,1444.15) | 1046.09(885.62,1232.13) | -0.96(-1.20,-0.72) |
| Protein-energy malnutrition | 1048.80(850.20,1281.41) | 1324.22(1116.47,1558.65) | 0.37(0.17,0.58) | 1097.08(886.45,1360.32) | 962.76(803.07,1150.80) | -0.97(-1.16,-0.77) |
| Iodine deficiency | 58.27(45.51,71.72) | 53.26(41.79,65.98) | -1.17(-2.02,-0.31) | 83.37(65.18,103.43) | 83.33(65.07,103.40) | -0.85(-1.69,0.00) |
| Vitamin A deficiency | 49428.14(42176.54,57025.67) | 30358.71(24238.10,37178.78) | -1.68(-2.12,-1.23) | 34238.28(29444.45,39519.21) | 21419.02(17728.64,25443.35) | -1.61(-2.08,-1.14) |
| Nepal |  |  |  |  |  |  |
| Nutritional deficiencies | 3187.93(2634.29,3826.34) | 2850.62(2429.07,3292.73) | -0.31(-0.37,-0.25) | 2688.91(2305.13,3215.33) | 2311.71(1961.52,2734.69) | -0.50(-0.69,-0.31) |
| Protein-energy malnutrition | 3125.89(2577.30,3757.98) | 2802.80(2383.86,3244.40) | -0.31(-0.37,-0.25) | 2600.31(2220.01,3120.94) | 2239.55(1892.24,2655.61) | -0.55(-0.74,-0.36) |
| Iodine deficiency | 62.04(48.01,79.02) | 47.82(37.04,61.44) | -0.40(-0.75,-0.05) | 88.60(68.07,115.55) | 72.16(54.90,91.15) | 1.10(0.28,1.94) |
| Vitamin A deficiency | 21567.47(17139.28,26359.51) | 4282.17(3208.14,5672.30) | -5.77(-5.91,-5.63) | 22562.64(19470.67,26275.86) | 6989.18(5738.14,8545.29) | -4.18(-4.37,-4.00) |
| Pakistan |  |  |  |  |  |  |
| Nutritional deficiencies | 2474.78(2074.22,2976.34) | 2304.75(1960.58,2698.47) | -0.98(-1.22,-0.74) | 2886.14(2417.33,3431.15) | 2575.77(2230.21,2987.11) | -0.79(-0.94,-0.63) |
| Protein-energy malnutrition | 2388.28(1986.28,2887.85) | 2235.71(1897.35,2627.78) | -0.99(-1.23,-0.74) | 2525.94(2068.23,3057.08) | 2140.38(1803.84,2538.51) | -1.09(-1.28,-0.91) |
| Iodine deficiency | 86.50(68.21,109.09) | 69.04(54.01,87.01) | -0.81(-0.88,-0.74) | 360.20(284.28,450.09) | 435.39(342.33,547.72) | 0.85(0.76,0.94) |
| Vitamin A deficiency | 24988.54(20630.79,30097.66) | 3158.79(2275.43,4398.17) | -7.10(-7.55,-6.65) | 16585.48(14100.58,19304.45) | 3903.39(3056.92,4919.74) | -4.99(-5.15,-4.82) |
| Central African Republic |  |  |  |  |  |  |
| Nutritional deficiencies | 1404.47(1247.80,1582.24) | 1161.13(1036.36,1309.73) | -0.54(-0.69,-0.38) | 1658.23(1394.93,2007.72) | 1377.89(1159.45,1666.36) | -0.67(-0.77,-0.57) |
| Protein-energy malnutrition | 1101.10(961.45,1271.54) | 985.66(871.08,1117.47) | -0.12(-0.40,0.17) | 1220.90(964.28,1560.77) | 1049.49(841.89,1327.73) | -0.30(-0.41,-0.19) |
| Iodine deficiency | 303.37(247.34,359.20) | 175.46(136.27,218.33) | -2.43(-3.06,-1.80) | 437.33(383.78,490.64) | 328.40(261.66,411.03) | -1.78(-2.08,-1.48) |
| Vitamin A deficiency | 64335.55(57373.22,71002.81) | 47754.69(40535.68,55034.11) | -1.03(-1.09,-0.97) | 27871.59(23632.03,32457.16) | 19728.46(16196.17,23540.99) | -1.16(-1.37,-0.95) |
| Democratic Republic of the Congo |  |  |  |  |  |  |
| Nutritional deficiencies | 1713.14(1560.90,1893.98) | 1371.73(1217.85,1539.60) | -0.78(-1.09,-0.47) | 2767.89(2259.01,3393.28) | 2269.33(1909.41,2713.10) | -0.91(-1.23,-0.59) |
| Protein-energy malnutrition | 1225.05(1093.80,1391.44) | 983.38(862.84,1120.09) | -1.00(-1.30,-0.69) | 1875.71(1386.63,2505.06) | 1557.10(1212.65,1964.78) | -0.95(-1.29,-0.60) |
| Iodine deficiency | 488.09(418.67,564.99) | 388.35(307.88,473.59) | -0.23(-0.60,0.14) | 892.17(814.18,984.85) | 712.23(576.54,863.21) | -0.85(-1.14,-0.56) |
| Vitamin A deficiency | 54104.60(46316.41,62192.14) | 38353.83(31390.94,45904.64) | -0.70(-1.21,-0.19) | 30441.11(25509.96,35548.40) | 21849.12(18137.63,26349.85) | -0.56(-1.21,0.10) |
| Burundi |  |  |  |  |  |  |
| Nutritional deficiencies | 2091.19(1808.59,2435.66) | 1369.22(1128.96,1648.51) | -2.07(-2.31,-1.83) | 1740.10(1514.99,2023.35) | 1268.92(1059.95,1519.74) | -1.09(-1.22,-0.96) |
| Protein-energy malnutrition | 1939.04(1660.53,2279.75) | 1279.34(1038.68,1557.33) | -2.06(-2.32,-1.81) | 1522.07(1297.20,1796.81) | 1135.12(924.80,1381.24) | -0.97(-1.11,-0.83) |
| Iodine deficiency | 152.15(131.61,172.51) | 89.88(69.96,116.89) | -2.13(-2.29,-1.98) | 218.03(192.21,246.29) | 133.80(104.39,175.57) | -2.04(-2.17,-1.90) |
| Vitamin A deficiency | 42238.83(35493.13,49754.65) | 25430.43(20152.35,31073.97) | -2.13(-2.34,-1.91) | 29299.65(24641.73,34274.33) | 18726.91(15487.74,22433.79) | -1.83(-2.06,-1.59) |
| Eritrea |  |  |  |  |  |  |
| Nutritional deficiencies | 3072.59(2592.02,3718.70) | 1587.68(1364.53,1860.93) | -2.15(-2.46,-1.83) | 2113.10(1809.45,2527.07) | 1292.41(1122.68,1485.58) | -1.90(-2.02,-1.78) |
| Protein-energy malnutrition | 3039.40(2561.02,3682.58) | 1564.36(1341.18,1837.44) | -2.16(-2.48,-1.85) | 2065.64(1761.53,2469.45) | 1259.86(1094.22,1454.61) | -1.92(-2.04,-1.80) |
| Iodine deficiency | 33.19(26.13,42.14) | 23.32(18.34,29.55) | -1.05(-1.24,-0.85) | 47.46(36.49,61.86) | 32.55(25.25,41.71) | -1.18(-1.37,-0.99) |
| Vitamin A deficiency | 61974.47(54259.85,68996.34) | 26862.85(20939.49,32972.13) | -2.86(-2.96,-2.75) | 41468.48(35989.22,47047.02) | 17269.64(14082.83,20478.90) | -2.93(-3.02,-2.84) |
| Ethiopia |  |  |  |  |  |  |
| Nutritional deficiencies | 2466.66(2154.64,2832.21) | 1802.87(1543.98,2114.39) | -1.02(-1.37,-0.67) | 2333.73(2006.47,2730.72) | 1779.85(1575.18,2017.32) | -1.12(-1.32,-0.92) |
| Protein-energy malnutrition | 1985.25(1684.09,2345.32) | 1445.10(1204.71,1748.77) | -1.18(-1.52,-0.85) | 1662.00(1378.77,2025.71) | 1328.22(1156.29,1531.94) | -1.16(-1.33,-0.99) |
| Iodine deficiency | 481.41(382.58,592.94) | 357.77(278.57,453.01) | -0.48(-1.11,0.14) | 671.72(539.62,824.07) | 451.63(354.23,572.78) | -1.04(-1.46,-0.63) |
| Vitamin A deficiency | 61910.95(55354.65,68899.24) | 27823.10(22280.83,34568.73) | -2.82(-3.19,-2.45) | 48784.58(43551.96,54344.96) | 20767.11(17007.97,24915.28) | -3.00(-3.36,-2.63) |
| Madagascar |  |  |  |  |  |  |
| Nutritional deficiencies | 2562.36(2168.39,3090.04) | 1699.06(1482.54,1937.56) | -1.36(-1.50,-1.22) | 2306.72(2036.11,2635.44) | 1570.48(1385.15,1781.81) | -1.44(-1.53,-1.35) |
| Protein-energy malnutrition | 2491.81(2097.35,3016.00) | 1623.90(1408.32,1864.40) | -1.42(-1.56,-1.28) | 2198.95(1926.60,2525.86) | 1455.19(1276.49,1663.21) | -1.55(-1.64,-1.46) |
| Iodine deficiency | 70.55(53.52,90.39) | 75.16(57.99,95.12) | 0.37(0.20,0.53) | 107.76(82.23,137.40) | 115.28(88.97,146.75) | 0.30(0.13,0.47) |
| Vitamin A deficiency | 46406.85(39047.99,53664.19) | 25767.58(20614.10,31719.26) | -2.10(-2.30,-1.89) | 30188.85(25323.66,34994.67) | 17943.65(14840.90,21450.70) | -1.75(-1.97,-1.52) |
| Malawi |  |  |  |  |  |  |
| Nutritional deficiencies | 1491.34(1177.55,1920.57) | 1149.20(973.35,1369.91) | -1.67(-1.88,-1.45) | 2103.38(1578.94,2758.03) | 2135.50(1712.82,2677.09) | -0.43(-0.69,-0.17) |
| Protein-energy malnutrition | 1378.17(1058.72,1814.88) | 1052.14(878.97,1260.92) | -1.68(-1.89,-1.47) | 1961.46(1439.58,2615.89) | 2011.92(1584.12,2540.16) | -0.36(-0.65,-0.08) |
| Iodine deficiency | 113.17(87.12,145.56) | 97.06(74.36,122.97) | -1.49(-1.85,-1.14) | 141.92(111.40,185.65) | 123.58(96.30,157.92) | -1.26(-1.54,-0.98) |
| Vitamin A deficiency | 56664.73(50247.61,63073.42) | 24560.27(19524.13,30889.17) | -3.15(-3.34,-2.96) | 48764.12(43351.91,53927.74) | 27349.17(23070.85,32155.75) | -2.12(-2.41,-1.83) |
| Mozambique |  |  |  |  |  |  |
| Nutritional deficiencies | 1623.25(1420.07,1896.93) | 930.39(803.80,1086.45) | -2.52(-2.72,-2.32) | 1232.08(1102.08,1386.94) | 667.97(585.89,760.78) | -2.59(-2.80,-2.37) |
| Protein-energy malnutrition | 1524.26(1325.11,1799.45) | 885.93(758.61,1042.83) | -2.52(-2.74,-2.30) | 1098.56(975.46,1253.28) | 609.41(529.55,703.02) | -2.59(-2.84,-2.34) |
| Iodine deficiency | 99.00(77.16,123.60) | 44.46(34.62,57.03) | -2.47(-2.85,-2.08) | 133.53(104.37,169.12) | 58.56(45.64,74.05) | -2.56(-2.92,-2.19) |
| Vitamin A deficiency | 76868.41(70468.91,82264.51) | 36007.45(29101.33,43652.99) | -2.85(-3.00,-2.71) | 50047.08(44304.10,55459.08) | 22189.86(18615.05,26243.78) | -2.97(-3.11,-2.84) |
| Rwanda |  |  |  |  |  |  |
| Nutritional deficiencies | 1499.89(1326.67,1686.93) | 1018.67(877.42,1180.70) | -2.14(-2.38,-1.91) | 1445.03(1246.27,1702.46) | 1123.75(965.49,1307.69) | -1.45(-1.70,-1.21) |
| Protein-energy malnutrition | 1368.55(1192.49,1551.04) | 921.36(786.18,1082.38) | -2.18(-2.41,-1.94) | 1252.32(1059.01,1496.35) | 977.70(823.65,1156.89) | -1.39(-1.68,-1.10) |
| Iodine deficiency | 131.34(100.75,162.29) | 97.30(74.76,123.90) | -1.84(-2.20,-1.47) | 192.71(152.20,241.48) | 146.05(112.52,186.35) | -1.85(-2.21,-1.49) |
| Vitamin A deficiency | 39823.63(32917.60,47302.51) | 16536.43(12695.92,20826.41) | -3.28(-3.64,-2.92) | 28193.08(23687.17,32862.85) | 12717.53(10347.67,15571.62) | -2.92(-3.31,-2.53) |
| Somalia |  |  |  |  |  |  |
| Nutritional deficiencies | 2756.17(2426.00,3153.10) | 2162.97(1915.05,2458.85) | -1.15(-1.38,-0.91) | 3216.52(2823.60,3671.44) | 2410.70(2128.61,2749.37) | -1.43(-1.61,-1.26) |
| Protein-energy malnutrition | 2183.56(1876.53,2564.63) | 1521.20(1322.79,1776.80) | -1.66(-1.96,-1.36) | 2468.11(2122.16,2889.82) | 1595.76(1358.32,1890.74) | -2.12(-2.38,-1.86) |
| Iodine deficiency | 572.62(454.95,700.08) | 641.77(516.22,776.37) | 0.40(0.28,0.52) | 748.42(605.68,916.42) | 814.94(668.32,982.48) | 0.22(0.11,0.34) |
| Vitamin A deficiency | 77680.01(71182.25,83125.73) | 72502.08(65646.71,78683.16) | -0.29(-0.33,-0.26) | 58377.26(53054.12,63870.71) | 54280.58(48351.29,59958.17) | -0.30(-0.35,-0.26) |
| United Republic of Tanzania |  |  |  |  |  |  |
| Nutritional deficiencies | 1202.23(1038.57,1397.67) | 767.53(650.27,908.81) | -2.11(-2.27,-1.95) | 1116.32(956.35,1315.59) | 805.82(696.93,939.58) | -1.36(-1.50,-1.21) |
| Protein-energy malnutrition | 1175.27(1010.69,1368.63) | 747.44(630.16,889.71) | -2.13(-2.29,-1.97) | 1078.62(922.95,1277.01) | 777.73(670.12,909.24) | -1.36(-1.51,-1.20) |
| Iodine deficiency | 26.96(20.94,34.62) | 20.09(15.79,25.16) | -1.27(-1.44,-1.09) | 37.70(29.39,48.30) | 28.10(21.98,36.45) | -1.37(-1.57,-1.17) |
| Vitamin A deficiency | 34681.15(28862.92,40840.61) | 14557.77(11325.89,18246.94) | -2.81(-3.02,-2.60) | 24613.29(20886.98,28427.96) | 13332.89(10944.62,15989.94) | -1.82(-2.08,-1.56) |
| Uganda |  |  |  |  |  |  |
| Nutritional deficiencies | 1202.12(998.86,1450.70) | 751.83(642.84,878.94) | -1.92(-2.05,-1.80) | 1119.47(909.50,1382.65) | 887.95(743.50,1047.86) | -1.03(-1.19,-0.88) |
| Protein-energy malnutrition | 1145.19(944.38,1391.98) | 713.18(604.33,838.43) | -1.94(-2.07,-1.81) | 1038.93(828.42,1296.76) | 834.15(691.59,989.49) | -0.99(-1.16,-0.82) |
| Iodine deficiency | 56.93(44.47,72.49) | 38.64(30.02,49.45) | -1.52(-1.61,-1.42) | 80.54(63.13,102.12) | 53.80(41.90,68.34) | -1.63(-1.73,-1.54) |
| Vitamin A deficiency | 36376.92(29950.97,43106.90) | 12353.74(9426.54,16028.11) | -3.94(-4.15,-3.73) | 27169.77(23256.24,31290.84) | 11228.04(9132.42,13418.53) | -3.19(-3.36,-3.03) |
| Benin |  |  |  |  |  |  |
| Nutritional deficiencies | 1969.26(1655.41,2369.26) | 1318.17(1143.81,1526.41) | -1.82(-1.98,-1.66) | 1564.64(1350.34,1850.78) | 1206.48(1065.30,1380.05) | -1.01(-1.11,-0.90) |
| Protein-energy malnutrition | 1924.79(1613.80,2326.51) | 1282.47(1107.80,1492.63) | -1.85(-2.01,-1.68) | 1492.22(1281.82,1779.92) | 1146.70(1007.37,1315.06) | -1.01(-1.13,-0.90) |
| Iodine deficiency | 44.47(34.54,56.72) | 35.70(28.14,44.94) | -0.89(-0.96,-0.82) | 72.42(55.88,93.08) | 59.78(46.66,76.30) | -0.83(-0.91,-0.74) |
| Vitamin A deficiency | 73065.05(66596.96,78711.05) | 37035.88(30279.35,44513.90) | -2.23(-2.30,-2.15) | 32922.59(27554.09,38452.28) | 17753.38(13909.94,21828.73) | -1.86(-2.08,-1.64) |
| Burkina Faso |  |  |  |  |  |  |
| Nutritional deficiencies | 2084.95(1838.56,2425.68) | 1934.06(1705.84,2186.76) | -0.34(-0.78,0.09) | 1641.39(1460.28,1870.29) | 1390.03(1236.65,1575.01) | -0.86(-1.23,-0.49) |
| Protein-energy malnutrition | 2022.97(1775.76,2361.86) | 1894.29(1665.06,2153.63) | -0.30(-0.75,0.15) | 1551.34(1375.83,1784.61) | 1331.16(1178.37,1517.73) | -0.82(-1.21,-0.43) |
| Iodine deficiency | 61.98(48.79,78.25) | 39.77(31.12,50.16) | -1.89(-2.03,-1.74) | 90.05(69.63,114.99) | 58.87(45.74,75.09) | -1.79(-1.91,-1.66) |
| Vitamin A deficiency | 79343.93(73273.53,83855.37) | 34743.61(28045.93,42184.87) | -3.12(-3.25,-2.99) | 40103.39(34517.80,45943.41) | 20743.03(17008.74,25399.01) | -2.38(-2.53,-2.23) |
| Chad |  |  |  |  |  |  |
| Nutritional deficiencies | 2269.24(1993.48,2620.04) | 1734.09(1504.90,2002.22) | -1.12(-1.22,-1.01) | 1976.38(1717.81,2322.57) | 1499.68(1336.26,1692.17) | -0.85(-1.03,-0.67) |
| Protein-energy malnutrition | 2185.23(1908.65,2544.61) | 1687.96(1456.41,1954.51) | -1.07(-1.18,-0.97) | 1857.58(1601.17,2217.65) | 1432.52(1268.80,1621.87) | -0.77(-0.95,-0.58) |
| Iodine deficiency | 84.01(66.33,105.03) | 46.13(35.55,58.98) | -2.45(-2.59,-2.30) | 118.80(92.73,150.15) | 67.16(52.27,85.84) | -2.39(-2.55,-2.24) |
| Vitamin A deficiency | 80950.35(75176.45,85135.62) | 46629.53(38674.43,54438.84) | -1.85(-1.90,-1.79) | 41203.81(35385.70,46981.65) | 22163.08(17982.82,27437.12) | -2.06(-2.27,-1.85) |
| C么te d'Ivoire |  |  |  |  |  |  |
| Nutritional deficiencies | 1386.25(1199.63,1603.74) | 1237.32(1075.74,1409.47) | -0.97(-1.13,-0.81) | 1189.97(1015.83,1403.69) | 998.54(878.09,1144.47) | -0.88(-0.96,-0.79) |
| Protein-energy malnutrition | 1354.05(1166.46,1570.27) | 1213.25(1050.97,1383.85) | -0.96(-1.12,-0.80) | 1066.17(893.78,1269.32) | 921.79(801.31,1066.98) | -0.75(-0.84,-0.67) |
| Iodine deficiency | 32.21(25.42,40.51) | 24.07(18.92,30.22) | -1.30(-1.49,-1.11) | 123.80(94.48,156.71) | 76.74(59.61,97.30) | -2.03(-2.22,-1.84) |
| Vitamin A deficiency | 43231.09(36934.09,49626.30) | 14540.48(10639.99,19329.71) | -3.51(-3.64,-3.38) | 33314.50(28639.36,37871.46) | 13920.56(11056.79,17264.84) | -2.79(-2.96,-2.63) |
| Gambia |  |  |  |  |  |  |
| Nutritional deficiencies | 2405.94(2033.33,2836.97) | 2017.71(1753.20,2332.42) | -0.65(-0.91,-0.38) | 1688.48(1492.68,1918.44) | 1225.40(1085.25,1396.45) | -1.22(-1.38,-1.06) |
| Protein-energy malnutrition | 2261.67(1883.44,2698.32) | 1916.75(1645.60,2226.37) | -0.59(-0.87,-0.31) | 1444.09(1263.20,1675.79) | 1045.58(911.24,1201.18) | -1.19(-1.36,-1.02) |
| Iodine deficiency | 144.27(111.13,183.54) | 100.96(78.13,127.91) | -1.55(-1.86,-1.23) | 244.39(192.61,309.94) | 179.82(139.75,231.51) | -1.39(-1.72,-1.07) |
| Vitamin A deficiency | 70475.17(63565.61,76074.44) | 29437.86(23279.23,36427.20) | -3.03(-3.09,-2.97) | 36157.86(31216.23,41572.22) | 16201.82(13102.36,19764.83) | -2.72(-2.78,-2.66) |
| Guinea |  |  |  |  |  |  |
| Nutritional deficiencies | 1439.92(1259.77,1670.37) | 1236.88(1078.53,1425.90) | -0.79(-1.05,-0.53) | 1597.97(1424.78,1804.27) | 1109.67(986.34,1240.33) | -1.29(-1.42,-1.17) |
| Protein-energy malnutrition | 1214.15(1042.12,1427.32) | 1148.43(989.14,1332.04) | -0.43(-0.72,-0.14) | 1290.03(1136.43,1500.82) | 974.23(861.06,1101.57) | -0.90(-1.00,-0.80) |
| Iodine deficiency | 225.77(178.11,277.55) | 88.45(68.22,113.09) | -3.64(-3.86,-3.41) | 307.94(245.49,380.66) | 135.44(104.38,171.25) | -3.33(-3.63,-3.03) |
| Vitamin A deficiency | 72519.06(65878.64,78189.22) | 33671.41(26861.77,40962.22) | -2.56(-2.71,-2.42) | 31808.65(26764.34,37419.46) | 16070.97(12912.18,19668.09) | -2.20(-2.28,-2.12) |
| Guinea-Bissau |  |  |  |  |  |  |
| Nutritional deficiencies | 1773.84(1529.07,2077.91) | 1323.16(1153.69,1525.45) | -1.41(-1.55,-1.27) | 1530.60(1353.00,1766.61) | 1073.53(944.80,1232.23) | -1.25(-1.37,-1.13) |
| Protein-energy malnutrition | 1617.03(1378.14,1911.65) | 1226.42(1061.16,1420.21) | -1.40(-1.55,-1.25) | 1188.10(1036.38,1413.41) | 889.30(771.32,1037.32) | -1.15(-1.28,-1.01) |
| Iodine deficiency | 156.81(125.51,193.74) | 96.74(74.48,125.14) | -1.56(-1.72,-1.39) | 342.50(273.63,429.03) | 184.22(143.42,233.72) | -1.69(-1.89,-1.49) |
| Vitamin A deficiency | 79800.07(74726.95,84102.21) | 38143.85(30995.80,45927.25) | -2.50(-2.56,-2.43) | 36282.56(31205.07,41407.85) | 17406.21(14058.37,21335.92) | -2.31(-2.42,-2.20) |
| Liberia |  |  |  |  |  |  |
| Nutritional deficiencies | 1385.55(1142.43,1687.27) | 1084.90(930.13,1253.81) | -1.47(-1.68,-1.26) | 1182.53(981.26,1470.74) | 845.59(741.75,964.54) | -1.63(-1.80,-1.45) |
| Protein-energy malnutrition | 1355.56(1111.18,1658.26) | 1057.24(904.26,1227.10) | -1.48(-1.69,-1.28) | 1134.76(930.47,1418.76) | 800.02(697.54,921.30) | -1.66(-1.83,-1.49) |
| Iodine deficiency | 29.99(23.45,38.11) | 27.67(21.43,35.33) | -1.08(-1.46,-0.70) | 47.77(36.90,61.10) | 45.57(35.65,58.29) | -1.02(-1.43,-0.62) |
| Vitamin A deficiency | 58162.55(50628.92,65209.75) | 21171.24(16294.13,26780.74) | -3.83(-4.06,-3.61) | 13365.02(10655.54,16698.36) | 5679.74(4363.05,7356.90) | -3.44(-4.03,-2.85) |
| Mali |  |  |  |  |  |  |
| Nutritional deficiencies | 2590.86(2276.47,2976.12) | 2128.81(1834.35,2480.33) | -1.28(-1.51,-1.04) | 1595.34(1413.68,1825.06) | 1609.11(1456.20,1788.19) | -0.04(-0.24,0.15) |
| Protein-energy malnutrition | 2542.90(2228.07,2929.48) | 2096.75(1805.87,2451.36) | -1.27(-1.51,-1.03) | 1516.59(1335.54,1745.98) | 1555.66(1403.81,1729.57) | 0.02(-0.18,0.23) |
| Iodine deficiency | 47.96(37.55,61.17) | 32.06(25.03,40.71) | -1.60(-1.70,-1.50) | 78.75(61.93,101.04) | 53.44(41.27,68.60) | -1.55(-1.65,-1.45) |
| Vitamin A deficiency | 77986.37(72785.69,82858.30) | 38781.33(31120.00,46392.77) | -2.54(-2.62,-2.46) | 39935.06(34331.08,45223.78) | 22468.31(18265.44,27082.32) | -2.03(-2.10,-1.95) |
| Niger |  |  |  |  |  |  |
| Nutritional deficiencies | 2080.37(1841.03,2410.27) | 1720.83(1536.66,1922.60) | -1.04(-1.17,-0.92) | 1883.83(1675.41,2151.37) | 1556.60(1410.62,1741.68) | -1.04(-1.19,-0.88) |
| Protein-energy malnutrition | 1957.26(1717.79,2283.68) | 1615.23(1432.26,1815.45) | -1.08(-1.21,-0.95) | 1717.57(1522.36,1985.73) | 1407.49(1264.48,1583.81) | -1.11(-1.29,-0.94) |
| Iodine deficiency | 123.10(99.28,149.78) | 105.59(81.95,135.97) | -0.53(-0.66,-0.39) | 166.26(132.99,206.21) | 149.11(115.16,191.54) | -0.31(-0.48,-0.14) |
| Vitamin A deficiency | 82826.28(77714.72,86821.40) | 55451.41(47393.95,63263.21) | -1.33(-1.43,-1.22) | 45012.24(39697.17,50973.95) | 31675.96(26792.42,36871.30) | -1.03(-1.12,-0.94) |
| Senegal |  |  |  |  |  |  |
| Nutritional deficiencies | 1458.38(1272.96,1661.23) | 1118.57(974.21,1275.18) | -0.80(-0.93,-0.67) | 1351.75(1200.16,1545.07) | 1095.76(968.69,1244.96) | -1.08(-1.23,-0.93) |
| Protein-energy malnutrition | 1355.81(1179.83,1562.17) | 1055.18(912.04,1206.60) | -0.72(-0.85,-0.59) | 1210.27(1055.62,1404.65) | 1005.20(881.06,1147.54) | -1.00(-1.17,-0.84) |
| Iodine deficiency | 102.57(84.68,123.09) | 63.39(49.66,82.61) | -2.01(-2.25,-1.76) | 141.48(116.17,166.39) | 90.57(69.69,114.35) | -1.78(-1.97,-1.58) |
| Vitamin A deficiency | 86672.96(83014.26,89461.48) | 20931.33(15777.27,27228.68) | -4.65(-5.16,-4.14) | 26235.55(21831.98,31278.03) | 7814.31(6214.31,9800.03) | -3.72(-4.02,-3.41) |
| Sierra Leone |  |  |  |  |  |  |
| Nutritional deficiencies | 1801.27(1506.38,2194.10) | 1367.52(1170.35,1633.90) | -1.12(-1.28,-0.97) | 1526.38(1254.74,1872.26) | 1136.14(987.49,1330.28) | -1.26(-1.37,-1.16) |
| Protein-energy malnutrition | 1747.16(1448.86,2144.10) | 1322.51(1126.10,1589.55) | -1.13(-1.28,-0.98) | 1436.92(1170.92,1784.51) | 1059.08(915.23,1257.05) | -1.29(-1.40,-1.19) |
| Iodine deficiency | 54.11(42.07,68.95) | 45.02(35.18,57.38) | -0.90(-1.28,-0.51) | 89.46(69.12,113.92) | 77.05(59.75,98.02) | -0.81(-1.22,-0.39) |
| Vitamin A deficiency | 72679.77(66493.77,77770.44) | 31458.19(24809.55,38310.90) | -2.87(-2.97,-2.76) | 30819.34(25653.47,35576.97) | 15365.35(12359.31,18766.08) | -2.26(-2.50,-2.02) |
| Togo |  |  |  |  |  |  |
| Nutritional deficiencies | 1714.33(1437.33,2059.66) | 1450.86(1229.04,1694.98) | -0.90(-1.09,-0.71) | 1403.01(1196.67,1663.98) | 1125.00(987.11,1282.96) | -1.10(-1.25,-0.95) |
| Protein-energy malnutrition | 1600.26(1327.39,1946.88) | 1379.65(1161.54,1622.64) | -0.82(-1.02,-0.61) | 1234.83(1031.75,1490.18) | 1012.90(877.64,1167.09) | -1.00(-1.14,-0.86) |
| Iodine deficiency | 114.07(98.67,131.66) | 71.21(55.76,92.60) | -2.21(-2.55,-1.88) | 168.19(148.85,189.50) | 112.09(87.01,143.04) | -1.92(-2.22,-1.62) |
| Vitamin A deficiency | 68855.72(61409.40,75378.73) | 26750.77(21003.64,32936.55) | -3.03(-3.15,-2.91) | 25317.33(20634.53,30493.23) | 11190.98(8959.08,13903.24) | -2.40(-2.61,-2.18) |
| South Sudan |  |  |  |  |  |  |
| Nutritional deficiencies | 2533.29(2209.65,2896.16) | 1734.50(1520.64,1979.28) | -1.70(-1.94,-1.47) | 2170.01(1874.01,2563.19) | 1689.27(1460.62,1981.61) | -1.11(-1.25,-0.97) |
| Protein-energy malnutrition | 2474.49(2150.45,2828.76) | 1671.28(1460.65,1918.12) | -1.77(-2.01,-1.52) | 2086.70(1794.55,2483.04) | 1598.75(1370.76,1897.93) | -1.18(-1.32,-1.03) |
| Iodine deficiency | 58.80(45.68,76.15) | 63.23(49.54,80.34) | 0.36(0.22,0.49) | 83.32(64.23,106.53) | 90.51(70.70,115.33) | 0.32(0.16,0.48) |
| Vitamin A deficiency | 45271.00(38257.93,52830.36) | 23506.34(18488.39,29472.26) | -2.45(-2.63,-2.27) | 30398.47(25448.70,35628.05) | 16804.96(13562.68,20165.97) | -2.11(-2.23,-1.99) |

**Table 3 Country‐specific age-standardized DALYs rate for nutritional deficiency (including Subcategories) and their estimated annual percentage changes (EAPC) in low-sociodemographic-index by sex group, 1990-2019. DALYs=disability-adjusted life-years.**

|  | male |  |  | female |  |  |
| --- | --- | --- | --- | --- | --- | --- |
|  | 1990 | 2019 | eapc | 1990 | 2019 | eapc |
| Low sdi |  |  |  |  |  |  |
| Nutritional deficiencies | 3312.50(2744.97,4031.80) | 1246.52(955.90,1594.60) | -3.56(-3.67,-3.46) | 3512.70(2614.43,4704.01) | 1577.45(1204.60,2013.94) | -2.87(-2.98,-2.77) |
| Protein-energy malnutrition | 2246.70(1784.95,2815.05) | 500.41(397.03,616.18) | -5.38(-5.57,-5.19) | 2176.07(1478.44,3205.48) | 549.51(429.34,716.24) | -4.87(-4.99,-4.74) |
| Iodine deficiency | 93.55(57.49,152.60) | 61.23(36.18,101.97) | -1.28(-1.52,-1.04) | 128.92(76.63,219.19) | 94.65(53.88,165.09) | -1.10(-1.27,-0.93) |
| Vitamin A deficiency | 86.45(59.39,121.28) | 42.03(28.61,58.77) | -2.55(-2.70,-2.40) | 60.13(40.89,84.53) | 33.87(22.90,47.53) | -1.96(-2.22,-1.71) |
| Papua New Guinea |  |  |  |  |  |  |
| Nutritional deficiencies | 752.56(553.46,991.21) | 561.20(386.08,784.26) | -0.78(-0.91,-0.65) | 1011.20(837.69,1229.77) | 877.03(606.78,1237.79) | -0.79(-0.86,-0.72) |
| Protein-energy malnutrition | 309.10(231.80,399.78) | 175.78(127.61,240.48) | -1.61(-1.90,-1.32) | 569.35(491.28,658.05) | 159.44(101.87,265.14) | -1.55(-1.82,-1.28) |
| Iodine deficiency | 3.93(2.25,6.18) | 1.57(0.87,2.65) | -3.27(-3.85,-2.69) | 0.99(0.44,1.98) | 2.57(1.49,4.19) | -2.70(-3.22,-2.17) |
| Vitamin A deficiency | 26.11(15.04,41.69) | 18.14(9.72,29.58) | -0.64(-0.95,-0.32) | 12.56(7.41,19.38) | 15.84(9.27,24.90) | -0.57(-0.89,-0.25) |
| Solomon Islands |  |  |  |  |  |  |
| Nutritional deficiencies | 1076.76(771.94,1470.55) | 656.29(468.41,918.76) | -1.64(-1.70,-1.57) | 7.77(4.66,12.05) | 921.37(681.12,1225.45) | -1.25(-1.33,-1.16) |
| Protein-energy malnutrition | 558.95(353.01,853.48) | 220.56(153.71,302.35) | -3.21(-3.37,-3.04) | 562.71(371.73,820.65) | 295.83(206.65,419.83) | -2.61(-2.80,-2.43) |
| Iodine deficiency | 2.61(1.58,4.05) | 1.67(0.99,2.64) | -1.40(-1.68,-1.11) | 47.90(35.68,61.89) | 2.70(1.62,4.22) | -0.82(-1.04,-0.60) |
| Vitamin A deficiency | 63.50(39.72,97.79) | 44.35(25.62,70.58) | -0.89(-1.09,-0.70) | 18.71(10.88,29.92) | 30.44(17.80,49.36) | -0.97(-1.23,-0.72) |
| Haiti |  |  |  |  |  |  |
| Nutritional deficiencies | 2910.72(1908.60,4321.74) | 1120.20(785.51,1567.13) | -2.79(-3.15,-2.43) | 2437.96(1479.88,3831.30) | 1332.35(951.93,1838.15) | -2.63(-2.94,-2.32) |
| Protein-energy malnutrition | 2236.50(1294.81,3646.07) | 546.20(316.98,903.83) | -4.23(-4.67,-3.80) | 34.51(19.34,56.26) | 434.28(249.15,734.83) | -4.99(-5.39,-4.58) |
| Iodine deficiency | 25.12(14.18,39.60) | 20.55(11.27,32.99) | -1.00(-1.21,-0.79) | 60.53(39.93,88.77) | 25.37(14.42,41.26) | -0.97(-1.17,-0.78) |
| Vitamin A deficiency | 44.98(26.99,68.38) | 27.41(15.91,44.85) | -1.67(-1.70,-1.64) | 48.48(30.39,70.88) | 24.83(15.63,37.94) | -1.14(-1.22,-1.07) |
| Yemen |  |  |  |  |  |  |
| Nutritional deficiencies | 1928.90(991.40,6533.48) | 858.56(605.00,1241.11) | -2.95(-3.14,-2.76) | 394.87(248.20,593.75) | 1467.73(977.04,2096.83) | -1.77(-2.05,-1.50) |
| Protein-energy malnutrition | 1313.24(456.00,5761.66) | 245.41(145.62,495.21) | -6.12(-6.31,-5.93) | 24.12(14.49,37.38) | 225.50(76.11,513.35) | -6.64(-6.78,-6.49) |
| Iodine deficiency | 64.58(40.88,95.33) | 59.09(35.56,89.23) | -1.71(-2.27,-1.15) | 23.53(13.23,37.99) | 77.37(46.61,119.30) | -1.26(-1.72,-0.79) |
| Vitamin A deficiency | 75.35(49.69,110.65) | 42.26(25.37,65.58) | -2.50(-2.77,-2.23) | 2616.10(1919.46,3455.82) | 30.04(18.49,46.12) | -2.73(-3.09,-2.36) |
| Afghanistan |  |  |  |  |  |  |
| Nutritional deficiencies | 879.08(567.99,1377.77) | 372.81(257.99,526.26) | -3.20(-3.67,-2.73) | 1460.39(906.99,2218.21) | 722.16(521.65,990.54) | -3.24(-3.56,-2.93) |
| Protein-energy malnutrition | 566.98(300.93,998.88) | 137.06(89.20,210.22) | -5.18(-5.92,-4.44) | 311.10(157.91,561.94) | 270.97(160.94,464.45) | -5.06(-5.57,-4.55) |
| Iodine deficiency | 46.87(30.45,68.93) | 43.20(27.63,64.08) | -1.08(-1.86,-0.30) | 61.86(39.16,91.00) | 61.77(39.73,91.80) | -0.88(-1.63,-0.12) |
| Vitamin A deficiency | 47.16(29.41,73.60) | 29.40(17.20,45.89) | -1.62(-1.97,-1.26) | 1030.06(793.51,1341.67) | 27.80(17.09,41.80) | -1.14(-1.56,-0.71) |
| Nepal |  |  |  |  |  |  |
| Nutritional deficiencies | 2855.22(2015.79,3886.70) | 720.57(506.59,1018.10) | -4.55(-4.85,-4.25) | 6017.60(4907.33,7188.43) | 1027.08(737.51,1415.93) | -4.13(-4.45,-3.82) |
| Protein-energy malnutrition | 1976.92(1252.84,2959.19) | 183.44(136.07,252.63) | -7.95(-8.21,-7.69) | 5006.81(3910.60,6103.70) | 231.80(155.03,340.87) | -7.71(-7.98,-7.44) |
| Iodine deficiency | 13.85(6.32,27.02) | 10.39(4.73,20.52) | -0.44(-0.82,-0.06) | 43.14(23.90,72.45) | 16.34(7.58,31.87) | 1.18(0.34,2.02) |
| Vitamin A deficiency | 35.53(20.75,56.30) | 8.30(4.54,13.90) | -5.50(-5.74,-5.25) | 48.97(31.65,72.80) | 13.19(7.87,20.84) | -3.71(-3.95,-3.47) |
| Pakistan |  |  |  |  |  |  |
| Nutritional deficiencies | 1560.96(1137.85,2099.10) | 1102.13(789.45,1496.25) | -1.13(-1.21,-1.05) | 1827.58(1175.76,2901.51) | 1588.86(1187.98,2122.89) | -0.98(-1.05,-0.90) |
| Protein-energy malnutrition | 564.44(351.62,846.58) | 271.58(188.68,377.94) | -2.07(-2.61,-1.52) | 1204.81(634.71,2212.77) | 304.13(205.79,437.04) | -2.47(-2.87,-2.06) |
| Iodine deficiency | 50.82(32.26,76.31) | 33.79(19.77,53.47) | -1.48(-1.71,-1.25) | 64.16(42.11,94.77) | 138.16(73.23,252.50) | 0.34(0.19,0.48) |
| Vitamin A deficiency | 62.66(40.41,91.48) | 18.25(11.66,27.06) | -4.29(-4.70,-3.87) | 40.19(24.68,59.24) | 13.74(8.85,20.02) | -3.06(-3.26,-2.86) |
| Central African Republic |  |  |  |  |  |  |
| Nutritional deficiencies | 2555.48(1676.92,3875.48) | 2213.81(1569.02,3056.89) | -0.11(-0.45,0.22) | 52.72(30.97,83.85) | 1940.27(1357.60,2759.75) | -0.20(-0.44,0.03) |
| Protein-energy malnutrition | 1775.98(1013.17,3057.11) | 1510.78(973.38,2327.65) | 0.04(-0.45,0.53) | 85.18(54.82,126.02) | 1169.56(679.86,1851.73) | -0.13(-0.54,0.27) |
| Iodine deficiency | 114.83(63.55,196.27) | 73.52(41.15,120.59) | -2.06(-2.56,-1.56) | 10.77(5.63,17.66) | 115.99(62.96,201.87) | -1.73(-1.98,-1.48) |
| Vitamin A deficiency | 117.92(75.70,178.49) | 89.85(55.20,133.67) | -1.05(-1.13,-0.97) | 14.60(8.85,21.70) | 54.38(34.70,82.85) | -1.06(-1.33,-0.80) |
| Democratic Republic of the Congo |  |  |  |  |  |  |
| Nutritional deficiencies | 2762.92(2011.67,3699.82) | 1165.79(834.94,1587.90) | -2.94(-3.46,-2.41) | 1764.18(940.10,3251.82) | 1270.21(853.90,1787.93) | -2.51(-2.86,-2.16) |
| Protein-energy malnutrition | 1881.05(1250.02,2797.00) | 496.75(283.48,768.42) | -4.32(-5.13,-3.50) | 504.23(251.13,928.88) | 387.37(212.85,686.85) | -4.31(-5.01,-3.60) |
| Iodine deficiency | 174.41(89.74,313.10) | 139.36(73.84,252.74) | -0.23(-0.74,0.28) | 117.25(79.70,166.00) | 245.49(126.24,461.69) | -0.81(-1.19,-0.43) |
| Vitamin A deficiency | 89.66(57.37,132.00) | 64.12(39.37,94.29) | -0.80(-1.29,-0.30) | 14.78(8.84,22.96) | 47.21(29.89,70.32) | -0.42(-1.01,0.17) |
| Burundi |  |  |  |  |  |  |
| Nutritional deficiencies | 4350.17(2644.64,6527.38) | 1516.80(1024.66,2286.67) | -3.67(-4.02,-3.31) | 1555.58(1067.45,2181.89) | 1363.94(952.54,1917.39) | -3.46(-3.70,-3.23) |
| Protein-energy malnutrition | 3661.68(2024.88,5762.87) | 1042.75(599.95,1759.21) | -4.36(-4.85,-3.88) | 554.96(277.58,937.68) | 822.35(471.42,1347.62) | -4.42(-4.79,-4.05) |
| Iodine deficiency | 69.19(41.56,108.30) | 39.23(22.29,63.77) | -2.29(-2.54,-2.04) | 127.84(62.98,243.06) | 50.82(28.76,87.75) | -2.27(-2.48,-2.05) |
| Vitamin A deficiency | 53.83(32.26,82.22) | 32.79(18.78,52.72) | -2.21(-2.49,-1.92) | 696.02(555.63,867.81) | 27.76(16.73,43.37) | -1.81(-2.10,-1.53) |
| Eritrea |  |  |  |  |  |  |
| Nutritional deficiencies | 10214.86(5496.40,15190.29) | 1680.57(1211.46,2236.74) | -5.71(-6.07,-5.35) | 3449.73(2293.79,4895.10) | 1712.53(1271.20,2330.67) | -4.67(-5.09,-4.25) |
| Protein-energy malnutrition | 9035.94(4482.30,13854.22) | 1066.91(688.68,1518.45) | -6.72(-7.08,-6.35) | 129.71(72.93,220.69) | 924.46(601.88,1372.42) | -5.98(-6.42,-5.53) |
| Iodine deficiency | 21.82(13.12,33.21) | 9.54(5.50,15.39) | -2.66(-2.98,-2.35) | 1665.11(1098.53,2331.65) | 13.47(7.92,21.52) | -2.40(-2.74,-2.06) |
| Vitamin A deficiency | 90.46(58.57,132.56) | 35.89(20.24,57.87) | -3.14(-3.24,-3.05) | 1034.46(562.65,1641.22) | 27.52(17.04,43.04) | -2.84(-2.90,-2.77) |
| Ethiopia |  |  |  |  |  |  |
| Nutritional deficiencies | 5433.00(3893.70,7134.82) | 1014.40(765.16,1299.07) | -6.33(-6.66,-5.99) | 17.54(9.60,29.06) | 1154.76(843.22,1509.86) | -5.32(-5.56,-5.08) |
| Protein-energy malnutrition | 4387.58(2962.56,5914.46) | 467.24(356.57,632.54) | -8.27(-8.79,-7.74) | 24.86(15.38,37.51) | 518.62(357.07,738.90) | -7.16(-7.55,-6.78) |
| Iodine deficiency | 185.37(104.03,322.62) | 114.13(59.82,209.52) | -1.18(-1.87,-0.50) | 36.46(22.94,55.89) | 139.88(71.56,263.37) | -1.60(-2.07,-1.13) |
| Vitamin A deficiency | 88.15(58.98,125.61) | 41.67(25.76,62.98) | -2.68(-2.97,-2.39) | 29.47(18.67,44.32) | 37.89(24.65,56.06) | -2.34(-2.62,-2.06) |
| Madagascar |  |  |  |  |  |  |
| Nutritional deficiencies | 5816.99(4875.14,6945.61) | 1685.92(1231.12,2228.79) | -4.03(-4.19,-3.87) | 1604.47(1057.63,2395.96) | 1936.98(1389.50,2704.27) | -3.68(-3.85,-3.51) |
| Protein-energy malnutrition | 4957.53(4028.00,6029.09) | 1127.06(757.72,1591.42) | -4.75(-4.92,-4.58) | 854.17(443.49,1568.00) | 1261.22(796.46,1943.68) | -4.41(-4.58,-4.23) |
| Iodine deficiency | 33.00(18.36,51.93) | 30.66(17.71,50.17) | -0.27(-0.46,-0.07) | 40.28(21.36,70.03) | 41.89(23.53,72.05) | -0.10(-0.28,0.08) |
| Vitamin A deficiency | 67.20(40.57,99.08) | 32.19(18.77,49.40) | -2.74(-2.95,-2.54) | 71.18(46.49,102.87) | 28.30(17.47,43.99) | -1.92(-2.19,-1.65) |
| Malawi |  |  |  |  |  |  |
| Nutritional deficiencies | 4047.51(2910.13,5669.86) | 1367.63(1041.43,1749.05) | -4.14(-4.42,-3.86) | 4273.95(3782.10,4754.37) | 1432.15(1061.49,1880.46) | -3.79(-4.09,-3.49) |
| Protein-energy malnutrition | 3233.98(2140.58,4810.33) | 763.77(564.23,1047.00) | -5.39(-5.70,-5.08) | 3418.61(2763.57,3914.82) | 583.07(396.56,839.75) | -5.67(-6.03,-5.32) |
| Iodine deficiency | 55.97(32.86,88.71) | 38.91(21.53,64.89) | -2.10(-2.46,-1.75) | 13.39(7.16,21.85) | 45.29(25.24,77.23) | -1.89(-2.21,-1.58) |
| Vitamin A deficiency | 87.88(57.25,127.11) | 39.00(22.91,62.29) | -3.16(-3.36,-2.97) | 20.12(12.21,29.81) | 43.07(26.69,65.35) | -2.16(-2.41,-1.91) |
| Mozambique |  |  |  |  |  |  |
| Nutritional deficiencies | 4906.81(3212.10,7316.96) | 1371.50(1037.06,1795.47) | -4.69(-4.86,-4.51) | 867.47(662.33,1124.65) | 1271.47(928.21,1711.25) | -4.48(-4.72,-4.25) |
| Protein-energy malnutrition | 3938.23(2334.88,6306.59) | 725.35(523.21,1016.53) | -6.23(-6.42,-6.03) | 1057.10(832.69,1347.78) | 581.93(355.40,929.92) | -6.40(-6.64,-6.16) |
| Iodine deficiency | 62.16(38.58,92.88) | 19.96(11.33,31.79) | -3.90(-4.22,-3.58) | 483.63(401.11,586.17) | 24.20(13.94,39.03) | -3.77(-4.03,-3.50) |
| Vitamin A deficiency | 118.49(77.41,168.27) | 54.10(32.67,83.71) | -2.96(-3.11,-2.81) | 14.23(7.59,23.16) | 39.99(24.88,61.34) | -2.60(-2.78,-2.43) |
| Rwanda |  |  |  |  |  |  |
| Nutritional deficiencies | 3038.45(2092.36,4313.22) | 894.20(650.02,1239.91) | -5.38(-5.96,-4.79) | 13.91(8.17,21.78) | 983.60(705.90,1339.03) | -4.67(-5.15,-4.20) |
| Protein-energy malnutrition | 2438.56(1518.87,3705.69) | 565.16(373.48,864.15) | -6.36(-7.08,-5.65) | 1927.49(1312.68,2717.12) | 514.60(312.54,815.99) | -6.11(-6.75,-5.46) |
| Iodine deficiency | 34.75(15.96,67.25) | 24.92(11.37,49.73) | -2.04(-2.44,-1.63) | 1276.94(791.68,2009.50) | 38.83(17.95,75.40) | -2.04(-2.43,-1.64) |
| Vitamin A deficiency | 41.19(23.60,63.88) | 15.87(8.72,26.61) | -3.69(-4.15,-3.23) | 23.62(13.34,39.05) | 17.02(10.02,26.44) | -2.81(-3.33,-2.30) |
| Somalia |  |  |  |  |  |  |
| Nutritional deficiencies | 7092.08(4107.30,11946.96) | 3345.93(2149.99,5367.25) | -2.12(-2.84,-1.40) | 54.96(32.01,86.65) | 3094.17(2146.70,4779.90) | -2.21(-2.89,-1.53) |
| Protein-energy malnutrition | 5897.35(3081.65,10649.63) | 2355.54(1246.12,4276.34) | -2.57(-3.40,-1.72) | 1151.50(695.48,1824.57) | 1787.09(993.43,3319.33) | -2.91(-3.83,-1.97) |
| Iodine deficiency | 227.01(130.59,384.87) | 247.34(138.44,431.34) | 0.27(0.11,0.43) | 80.38(42.61,147.68) | 300.16(165.25,529.73) | 0.16(0.00,0.32) |
| Vitamin A deficiency | 101.23(65.04,148.49) | 86.01(54.39,126.82) | -0.70(-0.76,-0.65) | 86.60(57.19,126.31) | 75.56(48.43,112.74) | -0.57(-0.62,-0.52) |
| United Republic of Tanzania |  |  |  |  |  |  |
| Nutritional deficiencies | 3122.04(2324.97,4112.58) | 1247.44(912.42,1660.97) | -3.57(-3.77,-3.37) | 730.37(531.47,997.47) | 1345.19(967.09,1796.80) | -3.14(-3.37,-2.91) |
| Protein-energy malnutrition | 2205.24(1504.93,3173.94) | 602.18(409.14,846.66) | -4.85(-5.10,-4.59) | 174.74(133.39,235.46) | 637.54(396.83,1011.05) | -4.23(-4.47,-3.99) |
| Iodine deficiency | 15.67(8.61,24.08) | 7.81(4.38,12.31) | -3.04(-3.39,-2.68) | 66.72(39.40,102.30) | 11.05(6.32,17.69) | -2.82(-3.16,-2.48) |
| Vitamin A deficiency | 65.03(40.11,95.06) | 27.50(16.56,42.67) | -2.80(-2.91,-2.69) | 10.50(6.37,15.79) | 26.84(16.52,40.18) | -1.58(-1.79,-1.36) |
| Uganda |  |  |  |  |  |  |
| Nutritional deficiencies | 3066.63(1945.06,5283.69) | 987.73(702.76,1320.85) | -4.07(-4.33,-3.81) | 429.81(306.68,584.17) | 895.28(653.43,1223.46) | -2.84(-3.04,-2.64) |
| Protein-energy malnutrition | 2464.01(1384.63,4608.48) | 623.18(406.99,901.59) | -4.89(-5.21,-4.58) | 18.48(10.00,29.86) | 428.48(260.76,688.66) | -3.92(-4.17,-3.66) |
| Iodine deficiency | 34.61(19.95,52.15) | 16.04(9.11,25.67) | -3.09(-3.25,-2.92) | 14.36(8.66,21.56) | 20.96(11.76,34.10) | -2.83(-2.98,-2.68) |
| Vitamin A deficiency | 50.26(29.87,76.03) | 18.77(10.76,29.67) | -3.55(-3.71,-3.39) | 287.75(180.77,428.82) | 18.75(11.66,28.62) | -2.72(-2.87,-2.57) |
| Benin |  |  |  |  |  |  |
| Nutritional deficiencies | 2837.53(1730.07,4814.01) | 773.49(551.44,1060.71) | -4.52(-4.80,-4.23) | 946.98(556.24,1503.95) | 945.92(649.33,1333.22) | -3.96(-4.29,-3.63) |
| Protein-energy malnutrition | 2350.07(1267.52,4270.28) | 358.23(226.92,559.33) | -6.46(-6.82,-6.10) | 443.02(154.89,964.02) | 362.38(195.95,673.11) | -6.27(-6.68,-5.86) |
| Iodine deficiency | 22.00(12.03,35.12) | 14.60(8.00,23.41) | -1.66(-1.78,-1.53) | 1.12(0.49,2.27) | 22.49(12.57,37.18) | -1.36(-1.45,-1.26) |
| Vitamin A deficiency | 113.51(73.20,169.62) | 66.13(39.81,101.19) | -1.91(-2.12,-1.69) | 16.77(9.64,27.35) | 42.15(26.09,62.62) | -1.29(-1.64,-0.94) |
| Burkina Faso |  |  |  |  |  |  |
| Nutritional deficiencies | 2378.94(1599.93,3555.41) | 1672.44(1146.37,2352.16) | -1.54(-2.18,-0.89) | 2724.63(1254.20,4704.38) | 1925.20(1217.63,2976.40) | -0.86(-1.22,-0.49) |
| Protein-energy malnutrition | 1611.58(922.53,2683.71) | 826.55(489.04,1412.36) | -2.69(-3.66,-1.71) | 1699.85(401.95,3543.50) | 981.35(470.77,1977.63) | -1.95(-2.54,-1.36) |
| Iodine deficiency | 33.60(19.33,52.59) | 16.95(9.45,27.29) | -2.75(-2.95,-2.55) | 82.77(52.00,130.43) | 22.98(13.03,37.51) | -2.47(-2.63,-2.30) |
| Vitamin A deficiency | 153.82(103.52,221.62) | 76.59(45.53,115.70) | -2.69(-2.87,-2.51) | 56.10(35.26,84.23) | 56.36(35.27,83.80) | -1.61(-1.85,-1.37) |
| Chad |  |  |  |  |  |  |
| Nutritional deficiencies | 5056.87(3345.05,7397.82) | 1471.56(1043.40,2001.34) | -4.43(-4.59,-4.26) | 2426.50(1145.12,4173.44) | 1521.38(1115.38,2027.28) | -4.12(-4.28,-3.96) |
| Protein-energy malnutrition | 4233.90(2503.58,6625.69) | 760.22(457.73,1204.22) | -6.04(-6.27,-5.81) | 1185.71(274.50,2731.16) | 733.15(477.04,1125.90) | -5.83(-6.07,-5.59) |
| Iodine deficiency | 43.15(24.61,66.82) | 19.42(10.80,31.35) | -3.19(-3.36,-3.03) | 24.30(14.16,38.71) | 25.44(14.25,42.00) | -2.95(-3.10,-2.80) |
| Vitamin A deficiency | 131.70(84.88,191.29) | 84.29(54.07,123.51) | -1.53(-1.60,-1.46) | 51.67(31.87,75.04) | 49.45(31.32,74.26) | -1.85(-2.07,-1.62) |
| C么te d'Ivoire |  |  |  |  |  |  |
| Nutritional deficiencies | 1397.06(1000.17,1904.24) | 779.97(528.53,1110.52) | -2.25(-2.52,-1.99) | 1309.92(915.00,1867.31) | 873.25(587.78,1231.24) | -2.29(-2.56,-2.03) |
| Protein-energy malnutrition | 768.79(468.69,1228.10) | 175.80(114.42,260.63) | -5.47(-5.82,-5.11) | 365.54(194.17,731.39) | 193.27(102.37,360.48) | -5.26(-5.56,-4.96) |
| Iodine deficiency | 12.63(6.96,20.05) | 9.21(4.99,15.13) | -1.46(-1.73,-1.19) | 49.56(33.32,70.89) | 26.02(14.58,44.60) | -1.87(-2.05,-1.68) |
| Vitamin A deficiency | 95.75(61.55,139.37) | 40.39(23.16,64.85) | -2.86(-3.01,-2.71) | 48.14(29.95,74.09) | 36.92(21.97,56.99) | -2.13(-2.32,-1.95) |
| Gambia |  |  |  |  |  |  |
| Nutritional deficiencies | 2250.75(1644.68,3118.22) | 1097.68(771.67,1537.82) | -2.83(-3.00,-2.66) | 1014.70(764.74,1309.87) | 1382.91(962.49,1907.32) | -2.17(-2.33,-2.00) |
| Protein-energy malnutrition | 1323.04(858.83,2043.80) | 308.95(175.84,477.13) | -5.39(-5.66,-5.11) | 450.86(338.57,606.17) | 279.62(149.69,536.05) | -5.29(-5.61,-4.97) |
| Iodine deficiency | 51.87(28.46,92.12) | 37.65(20.63,64.15) | -1.42(-1.70,-1.13) | 40.35(24.72,62.85) | 59.49(32.58,107.39) | -1.37(-1.68,-1.06) |
| Vitamin A deficiency | 139.63(93.08,198.96) | 61.72(35.93,95.56) | -2.92(-2.98,-2.85) | 20.66(12.73,32.18) | 43.84(28.17,66.12) | -2.33(-2.41,-2.25) |
| Guinea |  |  |  |  |  |  |
| Nutritional deficiencies | 3174.71(2108.25,4761.44) | 1255.22(833.14,1805.35) | -3.19(-3.31,-3.06) | 481.57(345.00,658.69) | 1428.20(960.15,2190.52) | -3.26(-3.39,-3.13) |
| Protein-energy malnutrition | 2524.78(1494.83,4016.17) | 695.88(386.27,1184.21) | -4.34(-4.54,-4.14) | 118.13(88.67,154.08) | 709.41(361.77,1367.48) | -4.72(-4.88,-4.56) |
| Iodine deficiency | 88.26(46.96,152.36) | 34.13(18.31,57.42) | -3.63(-3.86,-3.41) | 30.10(17.07,50.24) | 46.56(25.61,82.26) | -3.48(-3.77,-3.19) |
| Vitamin A deficiency | 114.24(73.65,167.64) | 63.10(39.14,96.48) | -1.92(-1.99,-1.86) | 19.71(12.28,29.36) | 39.47(24.03,60.38) | -1.53(-1.71,-1.35) |
| Guinea-Bissau |  |  |  |  |  |  |
| Nutritional deficiencies | 3624.99(2142.32,6340.98) | 1063.90(714.98,1509.88) | -4.25(-4.43,-4.08) | 3307.91(938.05,6534.55) | 1136.28(802.02,1567.62) | -3.56(-3.74,-3.38) |
| Protein-energy malnutrition | 2868.30(1469.14,5490.56) | 367.11(168.50,655.93) | -7.09(-7.28,-6.90) | 2622.74(429.50,5776.94) | 292.06(159.60,482.06) | -7.07(-7.28,-6.86) |
| Iodine deficiency | 62.69(35.49,104.38) | 37.10(20.43,62.20) | -1.71(-1.93,-1.49) | 35.77(23.20,52.43) | 61.83(33.77,110.11) | -1.81(-2.00,-1.61) |
| Vitamin A deficiency | 127.61(82.57,185.50) | 68.52(40.62,106.59) | -2.14(-2.16,-2.11) | 53.26(31.52,80.19) | 42.84(26.57,65.56) | -1.79(-1.90,-1.67) |
| Liberia |  |  |  |  |  |  |
| Nutritional deficiencies | 3626.22(2327.95,5526.29) | 750.04(529.18,1028.32) | -5.73(-6.06,-5.41) | 671.73(454.50,961.12) | 819.80(560.88,1182.03) | -5.33(-5.61,-5.05) |
| Protein-energy malnutrition | 3060.39(1845.92,4958.03) | 385.72(231.48,591.89) | -7.50(-7.87,-7.12) | 59.41(38.90,82.55) | 383.22(222.14,689.72) | -7.16(-7.49,-6.82) |
| Iodine deficiency | 16.92(9.55,26.34) | 11.58(6.50,18.75) | -2.66(-3.41,-1.91) | 66.80(41.19,106.45) | 19.41(11.34,31.17) | -2.19(-2.86,-1.52) |
| Vitamin A deficiency | 103.47(65.51,151.87) | 37.82(21.42,62.07) | -3.91(-4.13,-3.68) | 15.18(9.09,24.28) | 17.43(10.34,26.72) | -3.00(-3.52,-2.48) |
| Mali |  |  |  |  |  |  |
| Nutritional deficiencies | 4476.88(2898.53,6601.61) | 3235.46(2256.97,4570.17) | -1.98(-2.51,-1.44) | 850.14(616.28,1132.03) | 5442.08(3878.11,7424.50) | -0.46(-1.05,0.13) |
| Protein-energy malnutrition | 3592.95(2084.20,5698.93) | 2276.91(1392.14,3522.12) | -2.65(-3.32,-1.97) | 212.66(155.64,288.93) | 4163.90(2721.37,6062.41) | -0.68(-1.39,0.04) |
| Iodine deficiency | 26.71(15.34,40.74) | 13.73(7.66,22.06) | -2.57(-2.73,-2.41) | 2.60(1.51,4.24) | 21.11(11.93,34.00) | -2.31(-2.45,-2.17) |
| Vitamin A deficiency | 143.47(94.26,198.89) | 85.23(51.99,127.50) | -1.89(-1.97,-1.80) | 15.36(9.01,24.40) | 60.62(37.62,89.52) | -1.43(-1.52,-1.33) |
| Niger |  |  |  |  |  |  |
| Nutritional deficiencies | 3495.54(2155.92,5728.99) | 1079.90(739.09,1533.97) | -4.49(-4.74,-4.24) | 1095.51(816.74,1436.95) | 1395.79(946.76,2000.59) | -4.02(-4.28,-3.77) |
| Protein-energy malnutrition | 2713.67(1433.33,4877.63) | 464.46(273.31,792.81) | -6.50(-6.86,-6.14) | 365.92(265.09,488.66) | 540.17(279.93,962.62) | -6.21(-6.58,-5.84) |
| Iodine deficiency | 59.17(34.16,92.68) | 43.93(23.67,73.65) | -1.14(-1.37,-0.91) | 4.02(2.36,6.59) | 54.47(30.57,95.16) | -0.93(-1.15,-0.71) |
| Vitamin A deficiency | 142.43(93.43,202.23) | 99.54(62.15,147.23) | -1.18(-1.24,-1.13) | 49.18(30.44,73.16) | 77.67(48.49,113.36) | -0.88(-0.99,-0.78) |
| Senegal |  |  |  |  |  |  |
| Nutritional deficiencies | 2035.15(1434.60,2859.15) | 923.64(628.58,1290.28) | -2.89(-3.00,-2.77) | 709.31(503.43,972.40) | 1052.14(724.78,1518.50) | -2.18(-2.30,-2.06) |
| Protein-energy malnutrition | 974.37(552.85,1613.40) | 163.65(61.21,277.98) | -6.33(-6.64,-6.03) | 212.59(150.17,306.82) | 164.22(72.53,373.71) | -5.81(-6.11,-5.52) |
| Iodine deficiency | 38.54(21.46,63.73) | 24.08(13.36,40.20) | -1.98(-2.21,-1.75) | 0.67(0.28,1.30) | 31.25(17.33,52.81) | -1.80(-1.99,-1.61) |
| Vitamin A deficiency | 152.86(101.06,217.07) | 38.66(21.54,63.56) | -4.61(-5.05,-4.18) | 19.57(11.45,30.73) | 20.51(12.75,31.85) | -3.40(-3.63,-3.17) |
| Sierra Leone |  |  |  |  |  |  |
| Nutritional deficiencies | 3906.60(2485.05,5955.11) | 1551.75(1047.25,2248.07) | -3.49(-3.81,-3.16) | 1355.01(990.74,1818.43) | 1487.71(937.91,2325.22) | -3.16(-3.32,-3.00) |
| Protein-energy malnutrition | 3109.18(1770.74,5133.89) | 839.90(469.36,1455.76) | -4.81(-5.30,-4.32) | 648.50(418.69,989.12) | 796.93(377.57,1552.84) | -4.52(-4.79,-4.25) |
| Iodine deficiency | 27.60(14.95,43.24) | 20.81(11.66,32.97) | -1.25(-1.85,-0.64) | 3.53(2.18,5.49) | 29.99(16.52,49.34) | -1.18(-1.74,-0.62) |
| Vitamin A deficiency | 132.71(87.30,187.18) | 68.97(43.42,105.63) | -2.24(-2.36,-2.12) | 45.01(28.56,68.58) | 46.49(29.40,70.21) | -1.42(-1.64,-1.20) |
| Togo |  |  |  |  |  |  |
| Nutritional deficiencies | 1690.49(1161.60,2479.49) | 757.88(535.57,1060.40) | -3.21(-3.45,-2.96) | 826.54(607.42,1116.81) | 938.10(640.65,1330.99) | -2.71(-3.02,-2.40) |
| Protein-energy malnutrition | 1050.93(611.42,1789.53) | 191.33(110.95,278.65) | -6.56(-6.89,-6.23) | 249.65(183.00,331.82) | 189.91(109.23,328.70) | -6.58(-6.88,-6.29) |
| Iodine deficiency | 45.57(25.79,74.68) | 28.46(15.89,46.17) | -2.13(-2.41,-1.85) | 2.92(1.73,4.80) | 39.84(21.97,68.24) | -1.96(-2.23,-1.69) |
| Vitamin A deficiency | 142.82(93.16,212.65) | 55.47(32.50,86.19) | -3.08(-3.21,-2.95) | 15.35(8.80,24.81) | 34.87(20.83,53.67) | -1.95(-2.12,-1.79) |
| South Sudan |  |  |  |  |  |  |
| Nutritional deficiencies | 8040.48(4491.45,12035.60) | 1853.61(1278.24,2661.08) | -5.41(-5.69,-5.13) | 784.07(564.92,1070.80) | 2072.82(1383.53,3327.92) | -4.65(-4.83,-4.47) |
| Protein-energy malnutrition | 7121.48(3599.64,11080.11) | 1219.51(743.67,1929.00) | -6.37(-6.73,-6.02) | 137.85(97.71,190.11) | 1330.21(783.93,2528.92) | -5.62(-5.83,-5.40) |
| Iodine deficiency | 19.54(10.67,33.25) | 20.66(10.90,35.39) | 0.20(0.07,0.33) | 11.00(6.08,17.71) | 28.11(14.65,50.43) | 0.18(0.03,0.33) |
| Vitamin A deficiency | 69.17(43.77,105.10) | 33.92(20.21,53.22) | -2.69(-2.88,-2.50) | 3.18(1.65,5.47) | 32.23(19.71,48.30) | -1.95(-2.03,-1.86) |
